# Supplementary material for: Phenyl Derivatives Modulate the Luminescent Properties and Stability of CzBTM-Type Radicals
Source: Molecules. 2024 Jun 18;29(12):2900. doi: 10.3390/molecules29122900 (PMC11206717; doi:10.3390/molecules29122900)
Supplement: Supplementary file 1 [file molecules-29-02900-s001.zip › molecules-3045198-supplementary.pdf]

molecules

MDPI

Supplementary Materials

Phenyl Derivatives Modulate the Luminescent Properties and Stability of CzBTM-type Radicals

Quanquan Gou [1], Jiahao Guan [1], Lintao Zhang [1] and Xin Ai [1,*]

[1] School of Materials Science and Engineering, Collaborative Innovation Center of Information Technology, Collaborative Innovation Center of Marine Science and Technology, Hainan University, No 58, Renmin Avenue, Haikou 570228, China.

* Correspondence: aixin133@hainanu.edu.cn (X.A.)

Contents

1. Synthetic procedures and structure characterization……………………………………………………………….………..2

1.1 Sythetic procedures……………………………………………..……………………………………………………2

1.2 Structure characterization………..………………………………………………………….…..……………………6

2. Crystallographic data……………………………………………………………………………………………….………..11

3. Absorption and fluorescence spectra of radicals in various solution……………………………...…………………..13

4. Transient photoluminescence decay spectra………………………………………...…………….……...…………15

5. Theoretical calculations…………………………………………………………………………………...……………15

6. Electrochemical properties of radicals………………………………………………………………………..………18

7. Stability of radicals……..19

Appendix (Calculation data)………………………………………………………………………………………………….21

References……………………………………………………………………………………………..………………..81

1. Synthetic procedures and structure characterization.

1.1 Synthetic procedures.

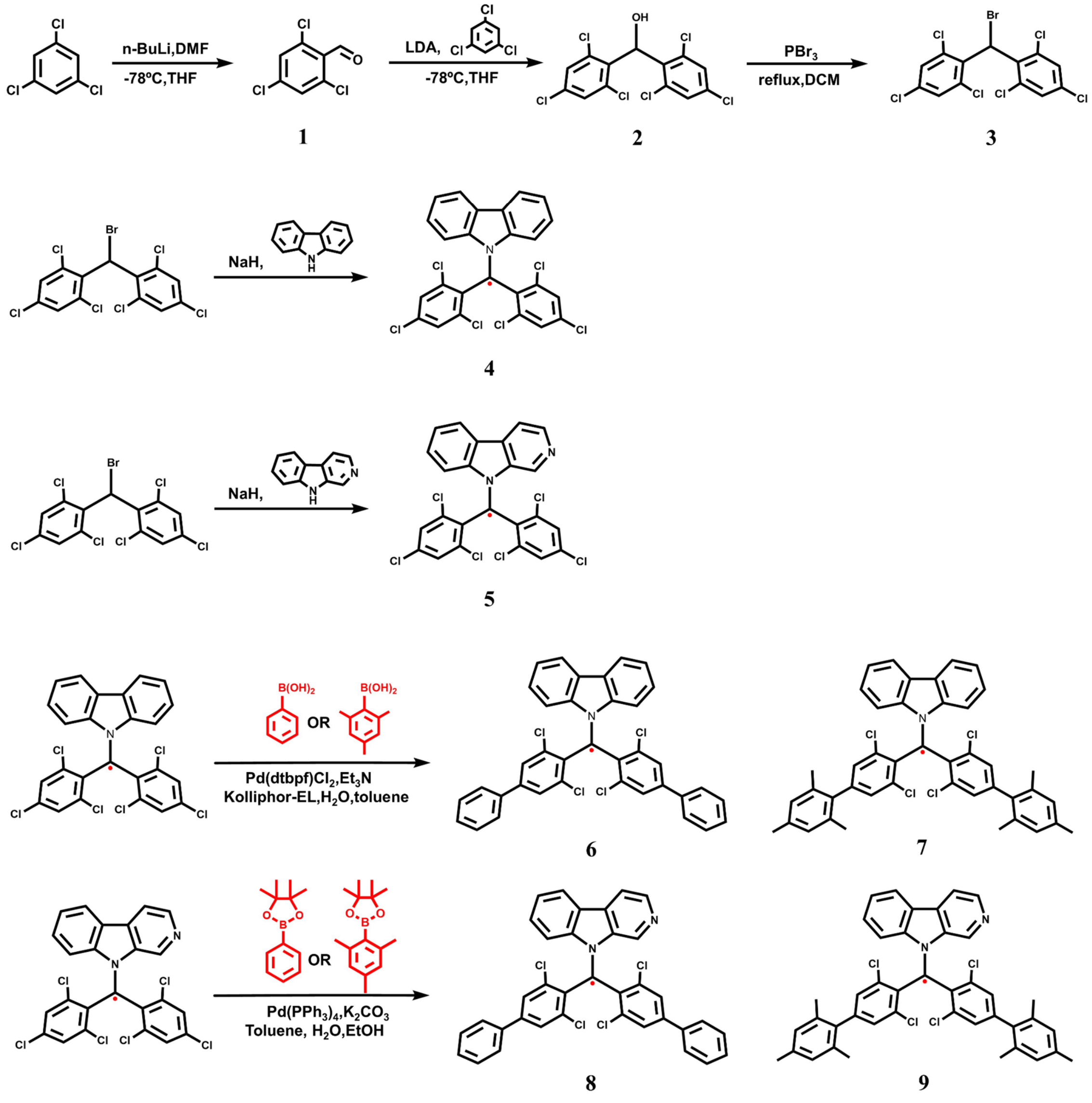

Figure S1. The synthetic routes of radicals

Synthesis of compound 1

Under argon atmosphere, 1,3,5-trichlorobenzene (10.0 g, 55.1 mmol) was mixed in anhydrous THF (100 mL). After n-BuLi in hexane (2.4 M, 23 mL) was added dropwise at -78 °C. The mixture was stirred at -78 °C for 0.5 h and the anhydrous DMF (7.44 mL, 107.3 mmol) was added slowly, then stirred for 1.5 h. After warmed slowly to room temperature, the mixture was poured into 3 M HCl solution and extracted with ethyl acetate by three times. The

combined organic layer was washed with sodium bicarbonate solution and dried over anhydrous sodium sulfate. After solvent was removed under reduced pressure and the crude product was purified by flash column chromatography using petroleum ether/dichloromethane (silica gel, 3:1, v/v) as eluent. White solid **compound 1** was obtained in 86% yield (10.0 g, 48 mmol). **^{1}H NMR** (400 MHz, Chloroform-*d*) δ 7.43 (s, 2H), 10.44 (s, 1H). **^{13}C NMR** (101 MHz, Chloroform-*d*) δ 127.78, 136.79, 138.34,186.68.

Synthesis of compound 2

Under argon atmosphere, 1,3,5-trichlorobenzene (9.5 g, 52.4 mmol) was mixed in anhydrous THF (100 mL). After lithium diisopropyl amide (LDA) in THF/n-hexane (2 M, 26.3 mL) was added dropwise at -78 °C. The mixture was stirred at -78 °C for 0.5 h and the compound **1** (10.0 g, 47.7 mmol) dissolved in anhydrous THF (60 mL) was added slowly, then stirred for 2 h. After warmed slowly to room temperature, the mixture was poured into saturated NH_4Cl solution and extracted with diethyl ether by three times. The combined organic solution was washed with sodium bicarbonate solution and dried over anhydrous sodium sulfate. After solvent was removed under reduced pressure and the crude product was purified by flash column chromatography used petroleum ether/dichloromethane (silica gel, 2:1, v/v) as eluent. White solid **compound 2** was obtained in 80% yield (15.0 g, 38 mmol). **^{1}H NMR** (400 MHz, Chloroform-*d*) δ 3.50 (s, 1H), 6.74 (s, 1H), 7.31 (s, 4H). **^{13}C NMR** (101 MHz, Chloroform-*d*) δ 72.96, 129.84, 134.49, 135.99.

Synthesis of compound 3 (HBTM-Br)

Compound 2 (4.0 g, 10.2 mmol) was mixed in anhydrous dichloromethane (100 mL). After phosphorus tribromide (2 mL, 21.3 mmol) dissolved in dichloromethane (10 mL) was added dropwise at 0 °C. Then the mixture warmed slowly to 45 °C and refluxed overnight. Next, cooled to room temperature, the mixture was poured into water and extracted with dichloromethane by three times. The combined organic solution was washed with brine and dried over anhydrous sodium sulfate. After solvent was removed under reduced pressure and the crude product was purified by flash column chromatography used petroleum ether (silica gel) as eluent. White solid **compound 3** was obtained in 80% yield (3.7 g, 8.2 mmol). **^{1}H NMR** (400 MHz, Chloroform-*d*) δ 7.08 (s, 1H), 7.37 (s, 4H). **^{13}C NMR** (101 MHz, Chloroform-*d*) δ 41.97, 130.01, 132.16, 134.91, 137.08.

Synthesis of compound 4 (CzBTM) and compound 4 (PyIDBTM)

(a). compound 4 (CzBTM): Under argon atmosphere, sodium hydride (60% in oil, 0.44 g,11.0 mmol) was dispersed in anhydrous DMF (90 mL). After carbazole (1.1 g, 6.6 mmol) dissolved in anhydrous DMF (10 mL) was added dropwise

and stirred for 30 min in the dark. Then **compound 3 (HBTM-Br)** (2.0 g, 4.4 mmol) was added and warmed slowly to 50 °C and stirred for 2 h. After that, the mixture was tardily cooled to room temperature and continue stirring for 3 h. And then the the mixture was poured into saturated NH_4Cl solution (400 mL). The precipitate was collected by suction filtration and purified by flash column chromatography used petroleum ether/dichloromethane (silica gel, 8:1, v/v) as eluent. Atropurpureus solid **compound 4 (CzBTM)** was obtained in 17% yield (0.4 g, 0.7 mmol). **LC-MS** (m/z): $[M]^+$ calcd. for $C_{25}H_{12}Cl_6N^{\cdot}$, 537.925; found, 537.909.

(b). compound 5 (PyIDBTM): The β-Carboline (0.9 g, 4.8 mmol) was added dissolved in anhydrous DMF (10 mL) was added dropwise and stirred for 30 min in the dark. Then **compound 3 (HBTM-Br)** (1.7 g, 3.8 mmol) was added and warmed slowly to 65 °C and stirred for 4 h. Used petroleum ether/ ethyl acetate (silica gel, 8:1, v/v) as eluent. Atropurpureus solid **compound 5 (PyIDBTM)** was obtained in 13% yield (0.25 g, 0.4 mmol). **MALDI-TOF** (m/z): $[M]^+$ calcd. for $C_{24}H_{11}Cl_6N_2^{\cdot}$, 538.90; found, 538.88.

Synthesis of compound 6 and 7

(a). compound 6 (Ph_2CzBTM): Under argon atmosphere, **compound 4 (CzBTM)** (0.4 g, 0.74 mmol), phenylboronic acid (0.2 g, 1.55 mmol) and Pd(dtbpf)Cl_2 (0.04 g, 0.06 mmol) were mixed degassed K-EL 2 wt%: toluene (9:1, v/v) emulsion (10 mL) in the dark. Next, the mixture was tardily warmed to 60 °C and degassed triethylamine (2 mL, 14 mmol) was added finally. After the reaction mixture was stirred for 12 h at 60 °C. And then the mixture was slowly cooled to room temperature, dichloromethane was added, and filtered on a celite pad. After solvent was removed under reduced pressure and the crude product was purified by flash column chromatography used petroleum ether/dichloromethane (silica gel, 24:1, v/v) as eluent. Black solid **compound 6 (Ph_2CzBTM)** was obtained in 30% yield (0.14 g, 0.22 mmol). **MALDI-TOF** (m/z): $[M]^+$ calcd. for $C_{37}H_{22}Cl_4N^{\cdot}$, 622.05; found, 622.05. **Elem. Anal.** calcd. for $C_{37}H_{22}Cl_4N^{\cdot}$ (%): C, 71.40; H, 3.56; N, 2.25. Found (%): C, 71.22; H, 3.41; N, 2.08. **IR (KBr)** 2924 (m), 2853 (w), 2369 (w), 2341 (w), 1635 (s), 1446 (m), 1386 (m), 1051(m), 880 (w), 757 (w), 565 (w), 478 (w).

(b). compound 7 (Mes_2CzBTM): Under argon atmosphere, **compound 4 (CzBTM)** (0.4 g, 0.74 mmol), 2,4,6-trimethylphenylboronic acid (0.25 g, 1.55 mmol) and Pd(dtbpf)Cl_2 (0.04 g, 0.06 mmol) were mixed degassed K-EL 2 wt%: toluene (9:1, v/v) emulsion (10 mL) in the dark. Next, the mixture was tardily warmed to 70 °C and degassed triethylamine (2 mL, 14 mmol) was added finally. After the reaction mixture was stirred for 48 h at 70 °C. And then the mixture was slowly cooled to room temperature, dichloromethane was added, and filtered on a celite pad. After solvent was removed under reduced pressure and the crude product was purified by flash column chromatography used

petroleum ether/dichloromethane (silica gel, 24:1, v/v) as eluent. Atropurpureus **compound 7 (Mes$_2$CzBTM)** was obtained in 23% yield (0.12 g, 0.17 mmol). **MALDI-TOF** (m/z): [M]$^+$ calcd. for $C_{43}H_{34}Cl_4N^\cdot$, 706.14; found, 706.14. **Elem. Anal.** calcd. for $C_{43}H_{34}Cl_4N^\cdot$ (%): C, 73.10; H, 4.85; N, 1.98. Found (%): C, 73.11; H, 4.59; N, 1.96. **IR (KBr)** 2922 (m), 2857 (w), 2346 (w), 1699 (m), 1633 (m), 1494 (m), 1447 (s), 1390(m), 1338 (w), 1192 (w), 1084 (w), 855 (w), 802 (w), 750 (m), 720 (m), 673(w).

Synthesis of compound 8 and 9

(a). compound 8 (Ph$_2$PyIDBTM): Under argon atmosphere, **compound 5 (PyIDBTM)** (0.2 g, 0.4 mmol), phenylboronic acid pinacol ester (0.2 g,0.9 mmol), Pd(PPh$_3$)$_4$ (0.04 g, 0.03 mmol), toluene (15 mL), ethanol (5 mL) and potassium carbonate solution (5 mL, 2 M) were added to a 250 mL two-necked flask. Then the mixture warmed slowly to 95 °C and stirred for 14 h. Next, cooled to room temperature, the mixture was poured into water and extracted with dichloromethane by three times. The combined organic solution was washed with brine and dried over anhydrous sodium sulfate. After solvent was removed under reduced pressure and the crude product was purified by flash column chromatography (Purified twice. First: petroleum ether/ ethyl acetate (silica gel, 8:1, v/v) as eluent; Second: petroleum ether/dichloromethane (silica gel, 1:1, lack solid solid **compound 8 (Ph$_2$PyIDBTM)** was obtained in 21% yield (0.05 g, 0.01 mmol). **MALDI-TOF** (m/z): [M]$^+$ calcd. for $C_{36}H_{21}Cl_4N_2^\cdot$, 623.04; found, 623.05. **Elem. Anal.** calcd. for $C_{36}H_{21}Cl_4N_2^\cdot$ (%): C, 69.36; H, 3.40; N, 4.49. Found (%): C, 69.55; H, 3.43; N, 4.21. **IR (KBr)** 3053 (w), 2925 (s), 2854 (m), 2360 (w), 1618 (w), 1573 (w), 1515 (m), 1458(s), 1431 (s), 1388 (m), 1326 (w), 1274 (w), 1195 (w), 1080 (w), 968 (w), 869(w), 800(m), 757(m), 694(m), 601(w).

(b). compound 9 (Mes$_2$PyIDBTM): Under argon atmosphere, **compound 5 (PyIDBTM)** (0.5 g, 0.9 mmol), 2,4,6-trimethylphenylboronic acid pinacol ester (0.7 g, 2.8 mmol), Pd(PPh$_3$)$_4$ (0.08 g, 0.06 mmol), toluene (15 mL), ethanol (5 mL) and potassium carbonate solution (5 mL, 2 M) were added to a 250 mL two-necked flask. Then the mixture warmed slowly to 95 °C and stirred for 48 h. Next, cooled to room temperature, the mixture was poured into water and extracted with dichloromethane by three times. The combined organic solution was washed with brine and dried over anhydrous sodium sulfate. After solvent was removed under reduced pressure and the crude product was purified by flash column chromatography (Purified twice. First (normal phase): petroleum ether/ ethyl acetate (silica gel, 8:1, v/v) as eluent; Second (reverse phase): water: acetonitrile (silica gel, 1:9, v/v) as eluent). Atropurpureus solid **compound 9 (Mes$_2$PyIDBTM)** was obtained in 15% yield (0.1 g, 0.1 mmol). **MALDI-TOF** (m/z): [M]$^+$ calcd. for $C_{42}H_{33}Cl_4N_2^\cdot$, 707.14;

found, 707.14. **Elem. Anal.** calcd. for $C_{42}H_{33}Cl_4N_2^{\cdot}$ (%): C, 71.30; H, 4.70; N, 3.96. Found (%): C, 71.05; H, 4.94; N, 3.87. **IR (KBr)** 2921 (m), 2854 (w), 2362 (w), 1618 (m), 1571 (w), 1506 (m), 1458(s), 1429 (s), 1384 (m), 1326 (w), 1269 (w), 1216 (w), 1189 (w), 1033 (w), 852(w), 800(m), 742(m), 609(w).

1.2 Structure characterization.

1.2.1 MALDI-TOF mass spectra

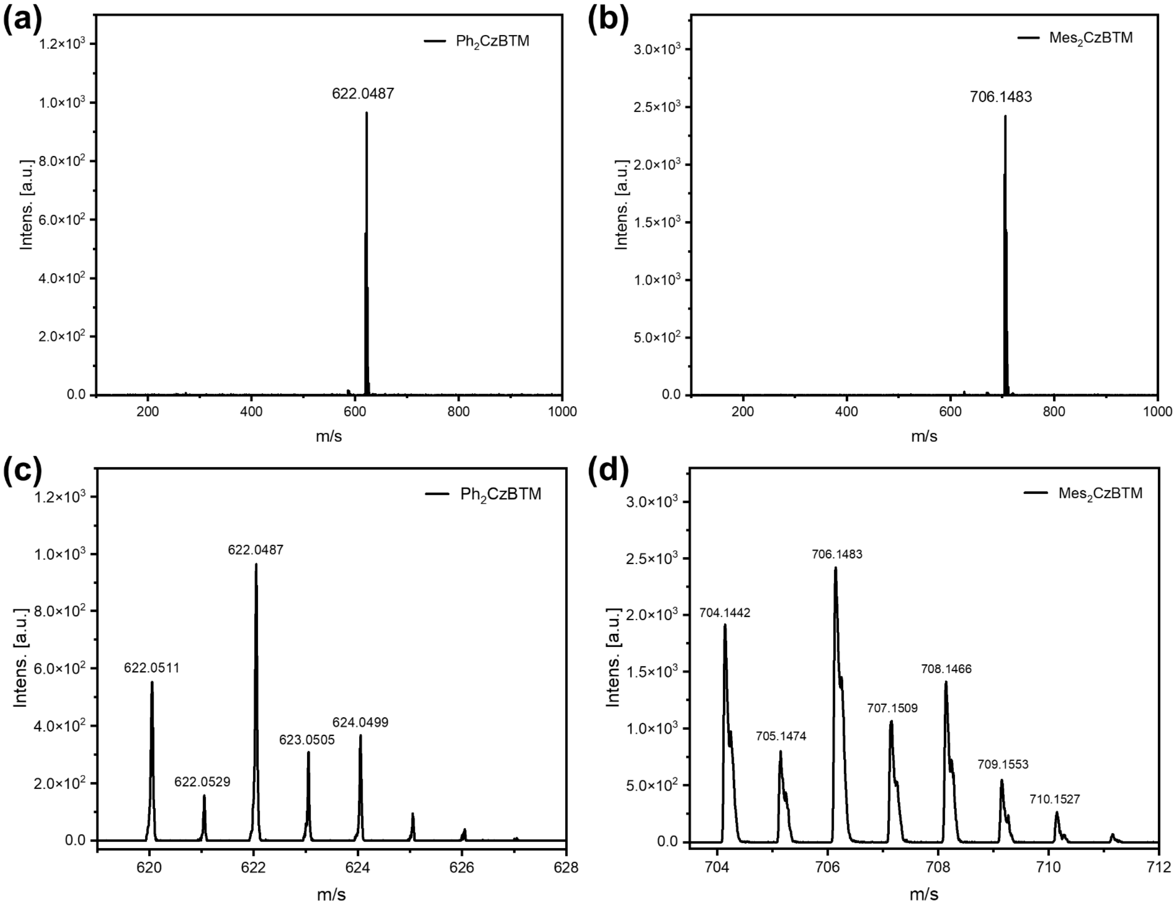

Figure S2. MALDI-TOF mass spectra of radicals: (a) (c) **Ph₂CzBTM**; (b) (d) **Mes₂CzBTM**.

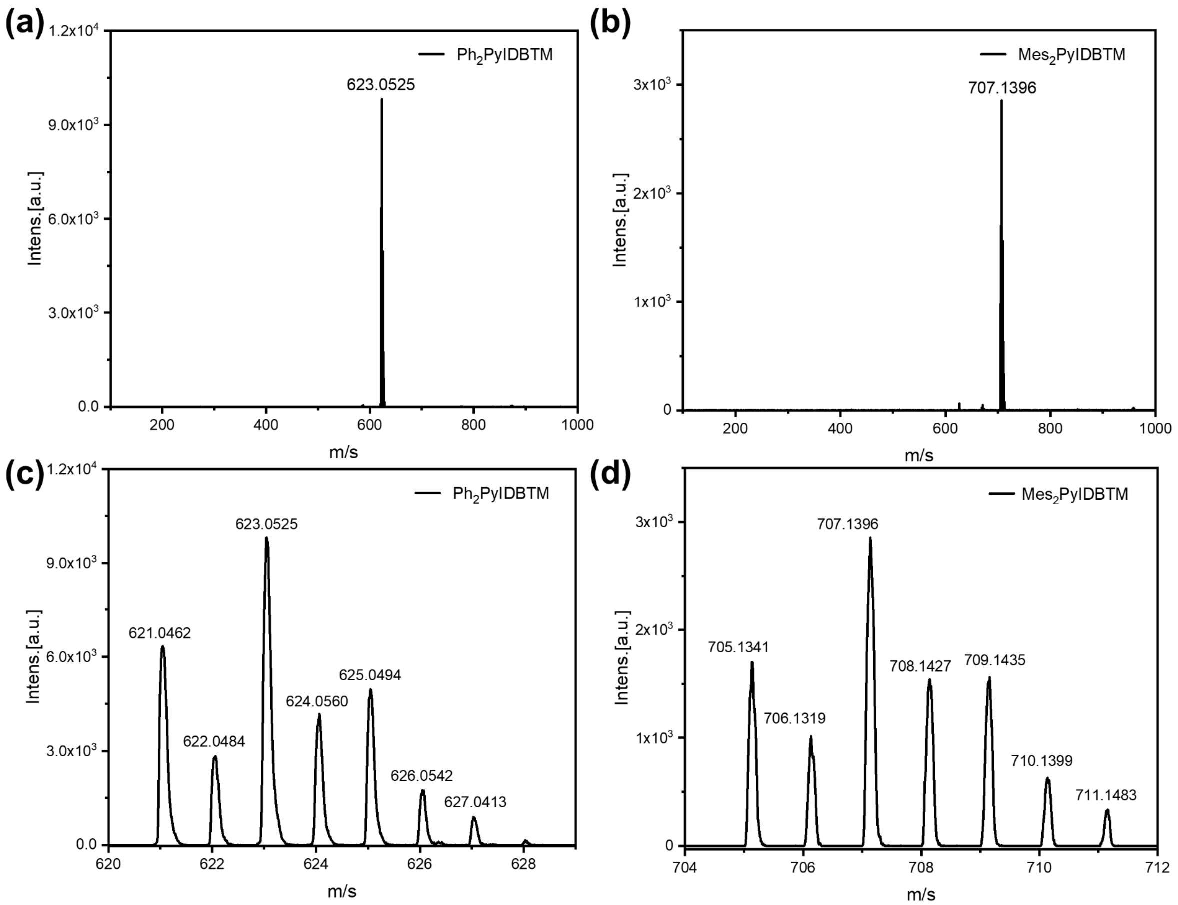

Figure S3. MALDI-TOF mass spectra of radicals: (a) (c) **Ph$_2$PyIDBTM**; (b) (d) **Mes$_2$PyIDBTM**.

1.2.2 FT-IR spectra

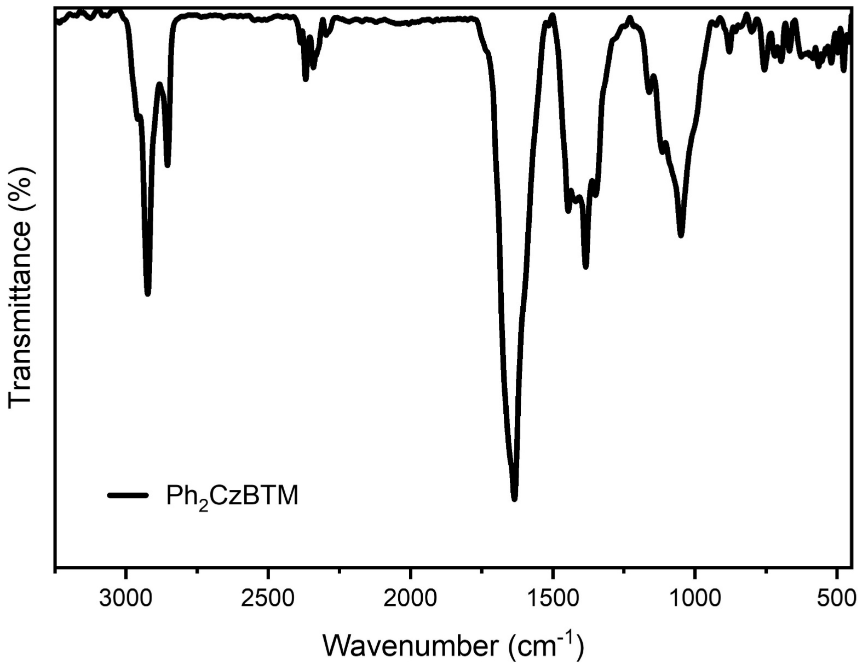

Figure S4. FT-IR spectra of **Ph2CzBTM.**

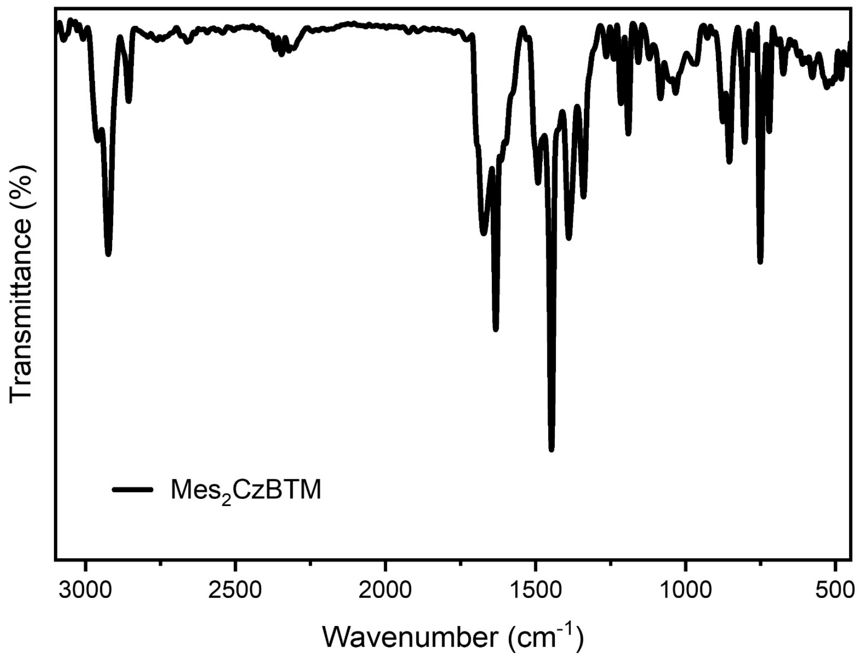

Figure S5. FT-IR spectra of **Mes2CzBTM.**

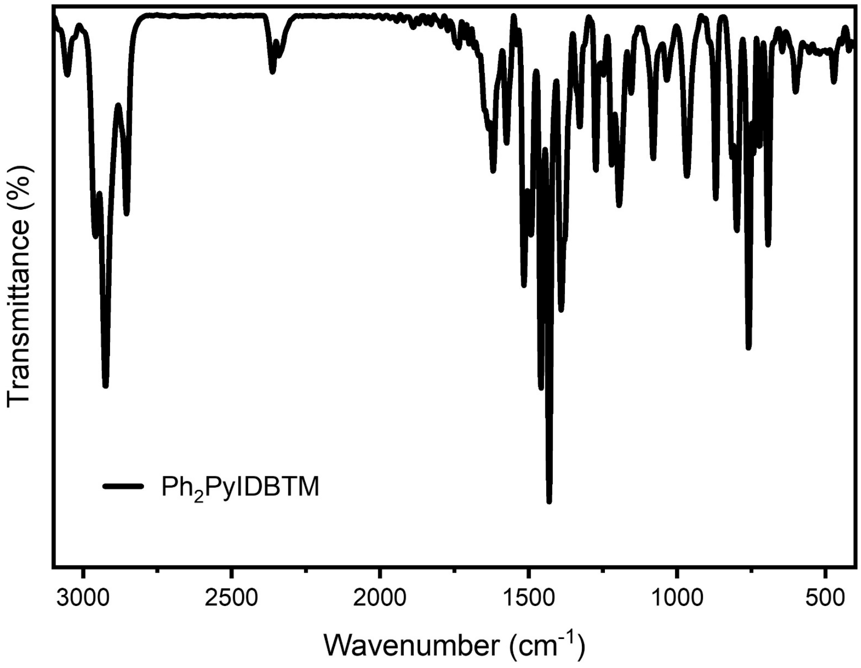

Figure S6. FT-IR spectra of **Ph2PyIDBTM.**

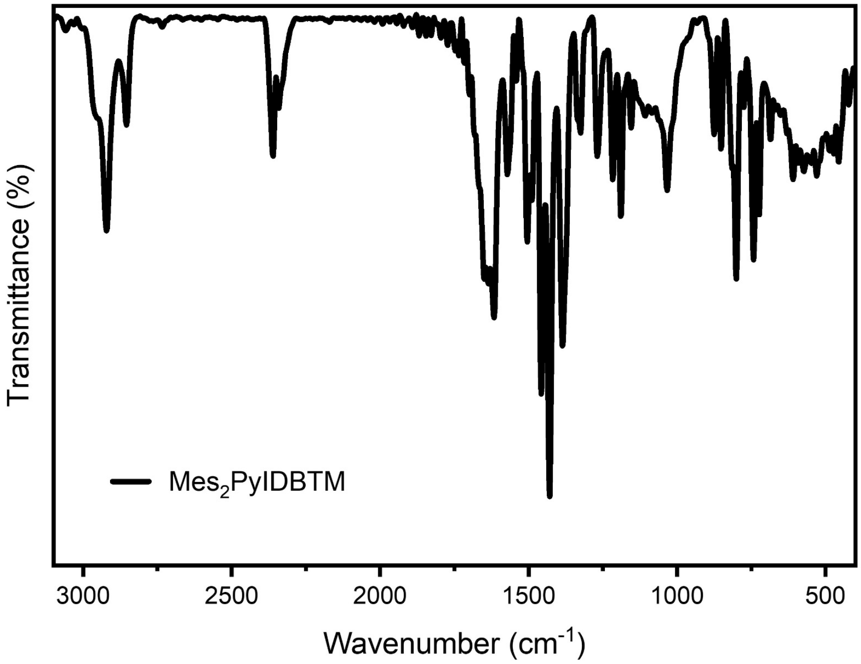

Figure S7. FT-IR spectra of **Mes2PyIDBTM**.

1.2.3 EPR spectra

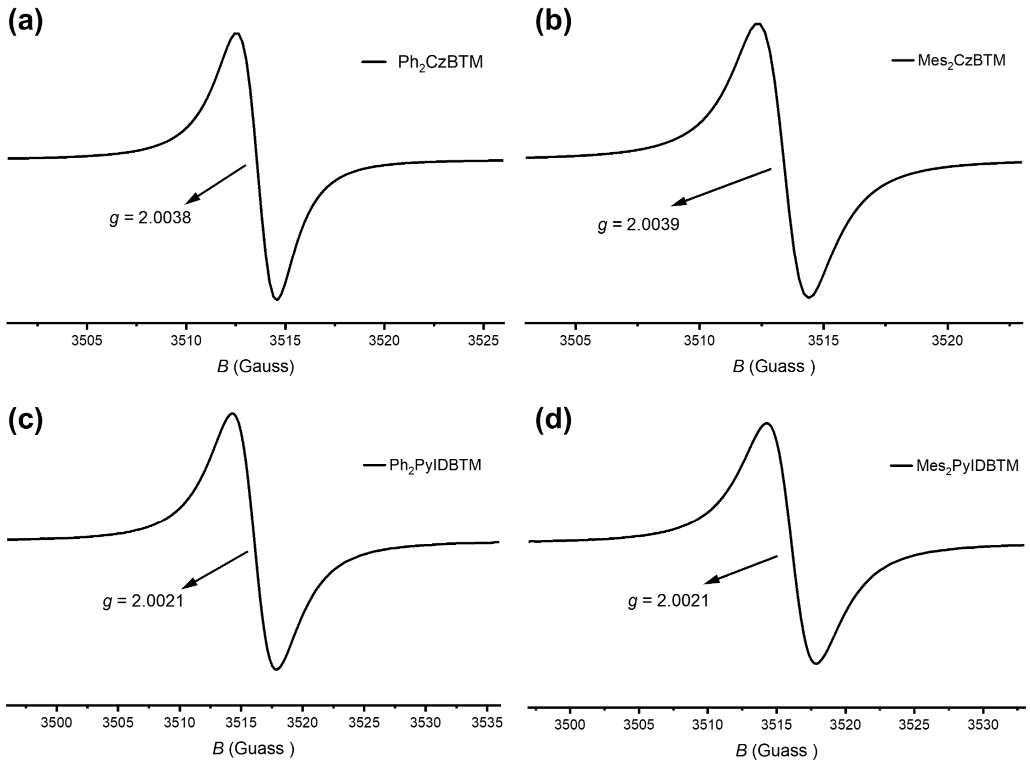

Figure S8. EPR spectra of radicals in powder form: (a) **Ph_2CzBTM**; (b) **Mes_2CzBTM**; (c) **Ph_2PyIDBTM**; (d) **Mes_2PyIDBTM**.

2. Crystallographic data.

Table S1. X-Ray Crystallographic Data of **Ph_2CzBTM** and **Mes_2CzBTM**.

Radicals	Ph_2CzBTM	Mes_2CzBTM
CCDC	2341126	2341148
Empirical formula	$C_{37}H_{22}Cl_4N$	$C_{44}H_{36}Cl_6N$
Formula weight	622.35	791.44
Temperature/K	100(2)	100.04(10)
Crystal system	monoclinic	triclinic
Space group	P21/c	P-1
a/Å	14.0383(4)	8.9349(2)
b/Å	7.8835(2)	12.8888(4)
c/Å	26.2239(8)	17.2990(4)
α/°	90	106.985(2)
β/°	98.265(3)	94.093(2)
γ/°	90	94.636(2)
Volume/Å3	2872.08(14)	1889.59(9)
Z	4	2
ρcalcg/cm3	1.439	1.391
μ/mm 1	0.442	0.489
F(000)	1276.0	818.0
Crystal size/mm3	0.3 × 0.2 × 0.15	0.26 × 0.25 × 0.2
Radiation	MoKα (λ = 0.71073)	Mo Kα (λ = 0.71073)
2Θ range for data collection/°	4.594 to 60.442	4.596 to 61.106
Index ranges	-17 ≤ h ≤ 17, -7 ≤ k ≤ 10, -32 ≤ l ≤ 36	-11 ≤ h ≤ 12, -17 ≤ k ≤ 17, -23 ≤ l ≤ 21
Reflections collected	25690	29171
Independent reflections	6952 [R_{int} = 0.0346, R_{sigma} = 0.0363]	9495 [Rint = 0.0291, Rsigma = 0.0343]
Data/restraints/parameters	6952/0/379	9495/0/466
Goodness-of-fit on F2	1.041	1.027
Final R indexes [I>=2σ (I)]	R_1 = 0.0383, wR_2 = 0.0898	R1 = 0.0382, wR2 = 0.0915
Final R indexes [all data]	R_1 = 0.0525, wR_2 = 0.0977	R1 = 0.0544, wR2 = 0.0997
Largest diff. peak/hole / e Å-3	0.37/-0.47	0.45/-0.33

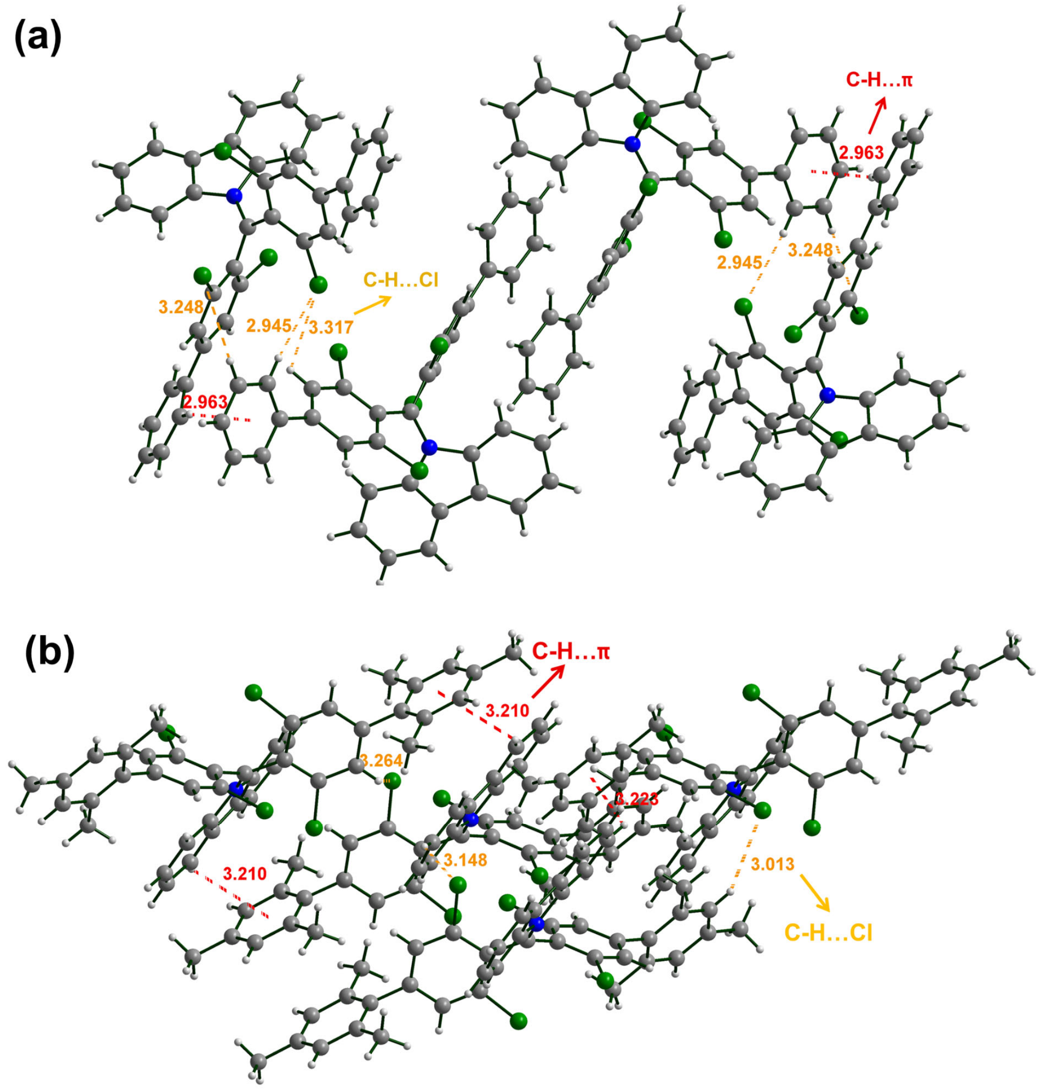

Figure S9. Intermolecular interactions: (a) **Ph₂CzBTM**; (b) **Mes₂CzBTM**.

3. Absorption and fluorescence spectra of radicals in various solutions.

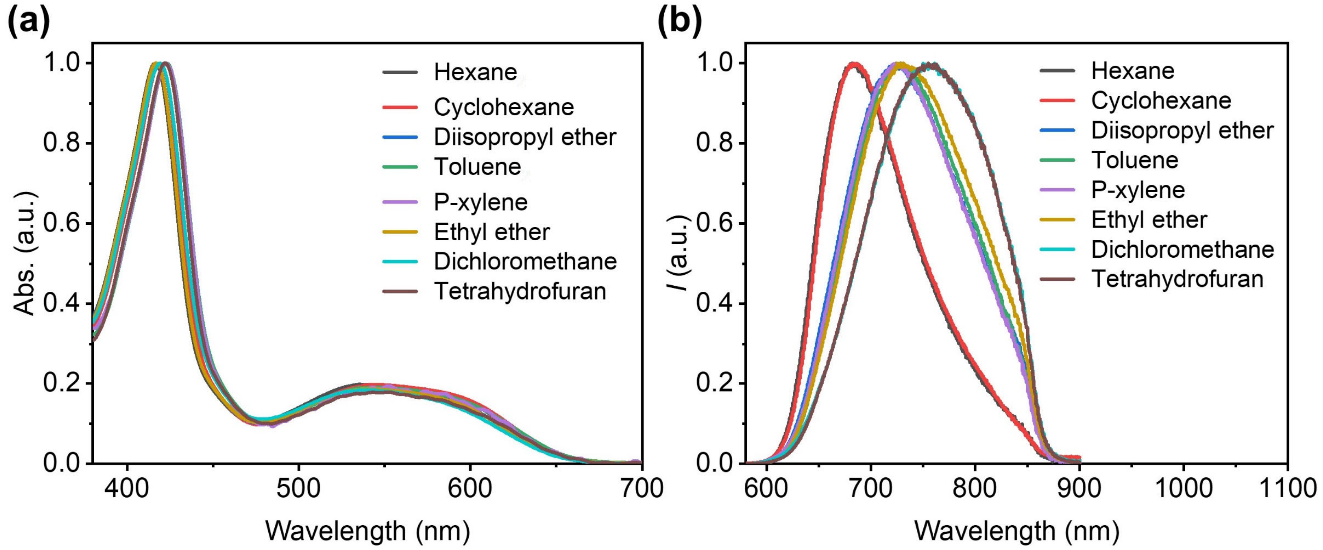

Figure S10. Absorption (a) and fluorescence (b) spectra of **Ph_2CzBTM** in various solutions at room temperature.

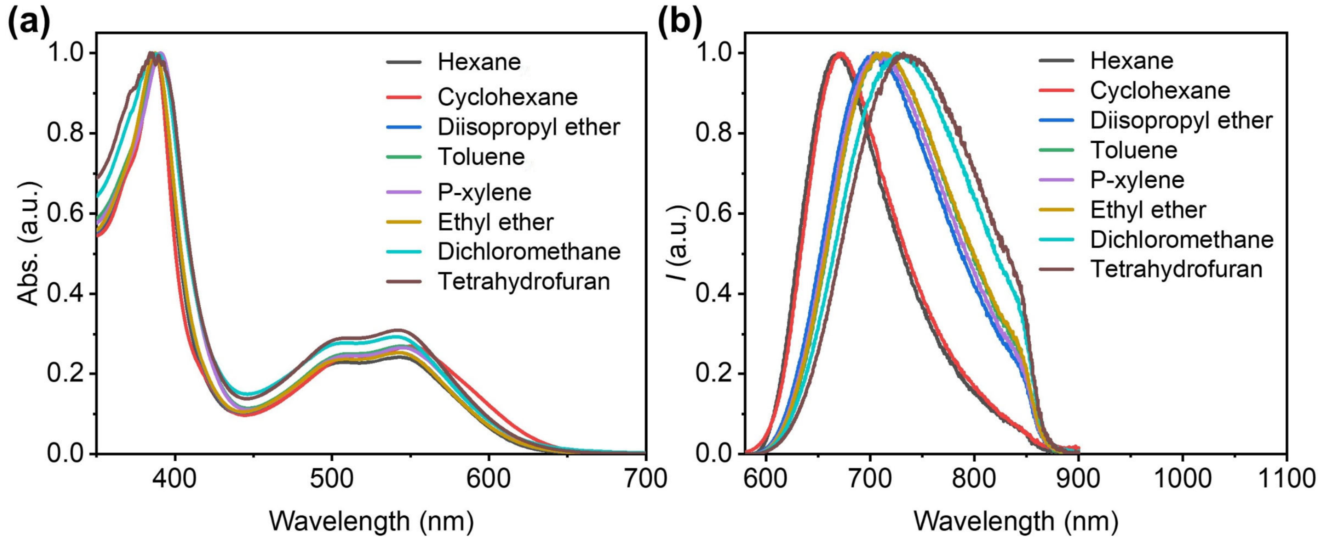

Figure S11. Absorption (a) and fluorescence (b) spectra of **Mes_2CzBTM** in various solutions at room temperature.

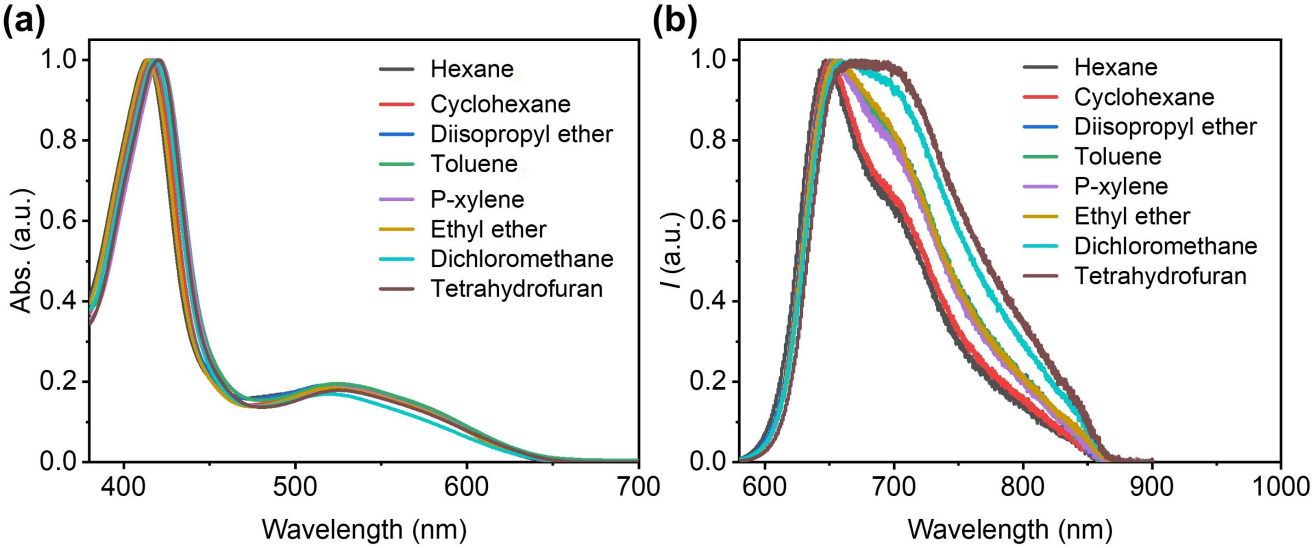

Figure S12. Absorption (a) and fluorescence (b) spectra of **Ph2PyIDBTM** in various solutions at room temperature.

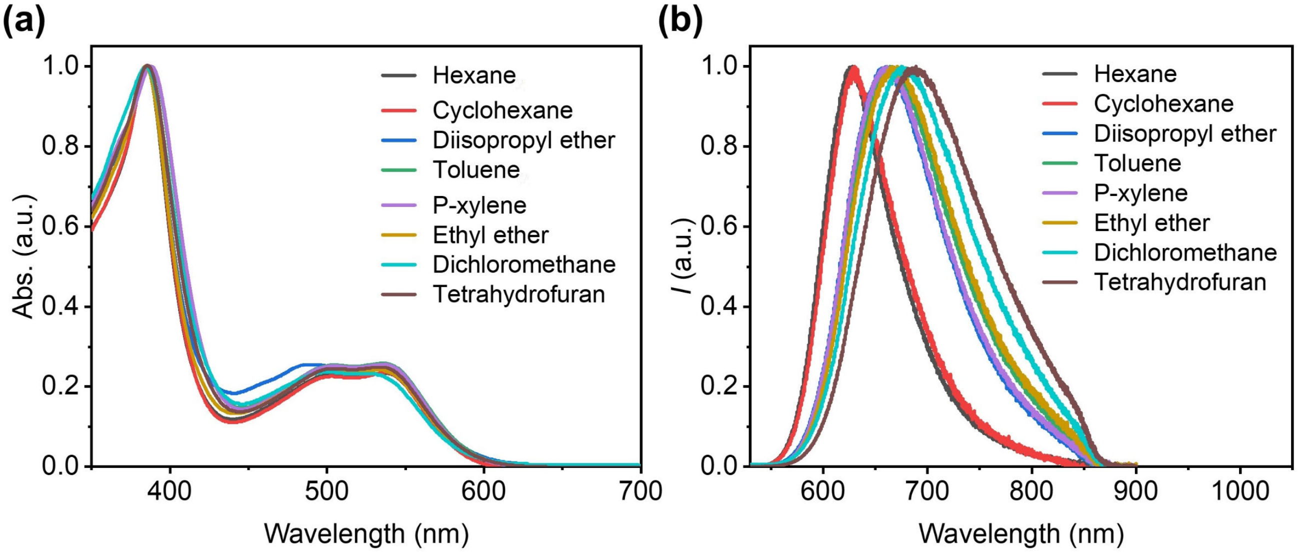

Figure S13. Absorption (a) and fluorescence (b) spectra of **Mes2PyIDBTM** in various solutions at room temperature.

4. Transient photoluminescence decay spectra.

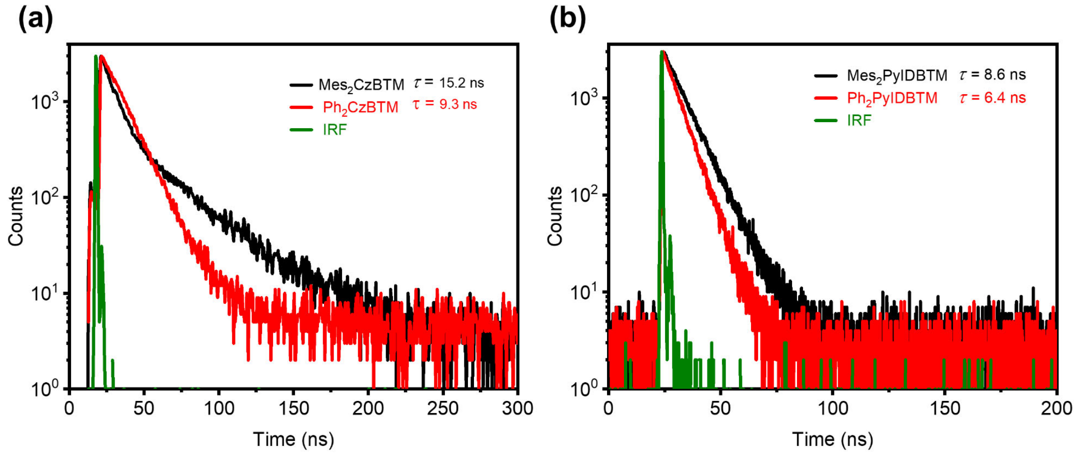

Figure S14. Transient photoluminescence decay spectra of radicals in cyclohexane at room temperature: (a) **Ph_2CzBTM** (red) and **Mes_2CzBTM** (black); (b) **$Ph_2PyIDBTM$** (red) and **$Mes_2PyIDBTM$** (black).

5. Theoretical calculations.

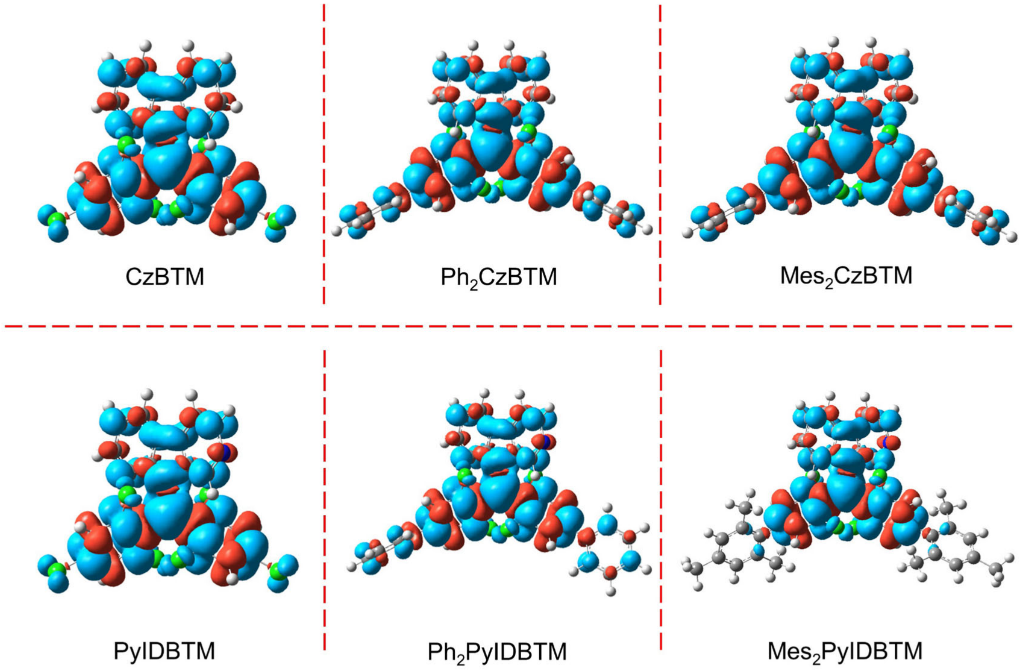

Figure S15. Spin density distribution of CzBTM, **Ph_2CzBTM**, **Mes_2CzBTM**, PyIDBTM, **$Ph_2PyIDBTM$** and **$Mes_2PyIDBTM$** by DFT calculations.

Table S2. The values of characteristic torsion angles in radical molecules in crystal structures and theoretical calculations.

Radicals	Angle	Torsion angle（°）		
		Crystal[b]	GS[c]	OES[d]
CzBTM[a]	α	40.2	43.2	58.0
	β	49.7	47.0	38.3
	γ	46.5	47.0	38.3
**Ph_2CzBTM**	α	47.7	43.9	66.3
	β	53.6	48.6	41.9
	γ	37.8	48.6	41.9
	θ	32.9	36.1	33.8
	δ	48.7	36.1	33.8
**Mes_2CzBTM**	α	35.6	43.9	66.8
	β	45.9	48.8	42.1
	γ	51.3	48.8	42.1
	θ	93.5	88.6	93.8
	δ	70.0	88.6	93.8
PyIDBTM	α	—	43.6	66.7
	β		48.8	41.8
	γ		48.9	42.4
**Ph_2PyIDBTM**	α	—	43.8	65.7
	β		48.8	42.2
	γ		48.2	41.0
	θ		36.1	33.7
	δ		36.0	33.0
**Mes_2PyIDBTM**	α	_	43.8	65.8
	β		49.1	42.3
	γ		49.0	41.7
	θ		88.2	92.9
	δ		87.8	92.0

[a] The data is sourced from the reference literature**Error! Reference source not found.**; [b] Crystallographic data; [c] Ground state; [d] Excited state; α is the torsion angle of the carbazole (β-carboline) group; β and γ are the torsion angles of the two dichlorobenzene groups; θ and δ are the torsion angles between the benzene ring (2,4,6-trimethylbenzene) and the dichlorobenzene.

Table S3. The bond lengths of radical molecules in crystal structures and theoretical calculations.

Radicals	Bond	Length（Å）		
		Crystal[b]	GS[c]	OES[d]
CzBTM[a]	N_1-C_{13}[e]	1.391	1.393	1.439
	C_{13}-C_{14}[f]	1.455	1.468	1.443
	C_{13}-C_{20}[f]	1.446	1.468	1.443
**Ph2CzBTM**	N_1-C_{13}[e]	1.399	1.395	1.465
	C_{13}-C_{14}[f]	1.466	1.466	1.442
	C_{13}-C_{26}[f]	1.447	1.466	1.442
	C_{17}-C_{20}[g]	1.396	1.483	1.480
	C_{29}-C_{32}[g]	1.483	1.483	1.480
**Mes2CzBTM**	N_1-C_{13}[e]	1.392	1.395	1.464
	C_{13}-C_{14}[f]	1.454	1.468	1.444
	C_{13}-C_{29}[f]	1.460	1.468	1.444
	C_{17}-C_{20}[g]	1.498	1.497	1.497
	C_{32}-C_{35}[g]	1.497	1.497	1.497
PyIDBTM	N_2-C_{12}[e]	—	1.395	1.465
	C_{12}-C_{13}[f]		1.467	1.444
	C_{12}-C_{19}[f]		1.468	1.443
**Ph2PyIDBTM**	N_2-C_{12}[e]	—	1.397	1.466
	C_{12}-C_{13}[f]		1.466	1.443
	C_{12}-C_{25}[f]		1.465	1.440
	C_{16}-C_{19} [g]		1.483	1.480
	C_{28}-C_{31}[g]		1.482	1.479
**Mes2PyIDBTM**	N_2-C_{12}[e]	—	1.397	1.465
	C_{12}-C_{13}[f]		1.467	1.444
	C_{12}-C_{28}[f]		1.468	1.444
	C_{16}-C_{19}[g]		1.497	1.497
	C_{31}-C_{34}[g]		1.497	1.497

[a] Data sourced from the reference literature**Error! Reference source not found.**; [b] Crystallographic data; [c] Ground state; [d] Excited state; [e] Bond length between the carbazole N_1 (β-carboline N_2) and the central carbon atom; [f] Bond lengths between the central carbon atom and the two carbon atoms connecting to dichlorobenzene; [g] Bond lengths between the two carbon atoms connecting the benzene ring (2,4,6-trimethylbenzene) and the dichlorobenzene.

Table S4. The parameters corresponding to the D_1 transition in the TD-DFT calculation results of radicals.

Radicals	Orbital excitation contribution	Transition energy (eV)	*f*
CzBTM	134β →136β (0.99)	2.19	0.09
**Ph2CzBTM**	159β →160β (0.98)	2.21	0.10
**Mes2CzBTM**	183β →184β (0.99)	2.27	0.09
PyIDBTM	135β →136β (0.99)	2.27	0.09
**Ph2PyIDBTM**	159β →160β (0.98)	2.28	0.10
**Mes2PyIDBTM**	183β →184β (0.99)	2.36	0.09

6. Electrochemical properties of radicals.

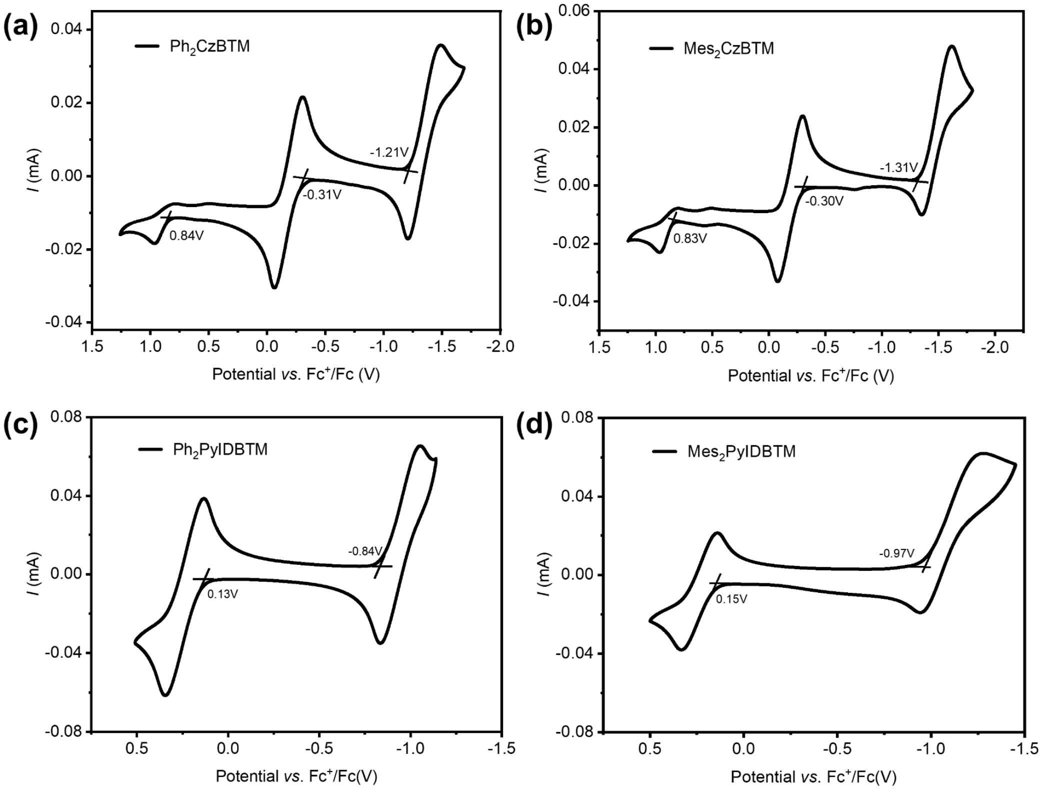

Figure S16. Electrochemical properties of radicals: (a) **Ph2CzBTM**; (b) **Mes2CzBTM**; (c) **Ph2PyIDBTM**; (d) **Mes2PyIDBTM**.

Table S5. Redox potential of free radicals and their corresponding orbital energy levels.

Radicals	E_{ox} (V)	E_{red} (V)	α-SOMO (eV)		β-SUMO (eV)	
			Calculated	Experimental	Calculated	Experimental
CzBTM[a]	″0.06	″0.92	″4.99	″4.74	″3.01	″3.88
**Ph2CzBTM**	″0.31	″1.21	″4.67	″4.49	″2.74	″3.59
**Mes2CzBTM**	″0.30	″1.31	″4.71	″4.50	″2.67	″3.49
PyIDBTM[b]	0.03	″1.03	″5.15	″4.83	″3.13	″3.77
**Ph2PyIDBTM**	0.13	″0.84	″4.82	″4.93	″2.86	″3.96
**Mes2PyIDBTM**	0.15	″0.97	″4.68	″4.95	″2.78	″3.83

[a][b] Data sourced from the reference literature**Error! Reference source not found.**[1].

7. Stability of radicals.

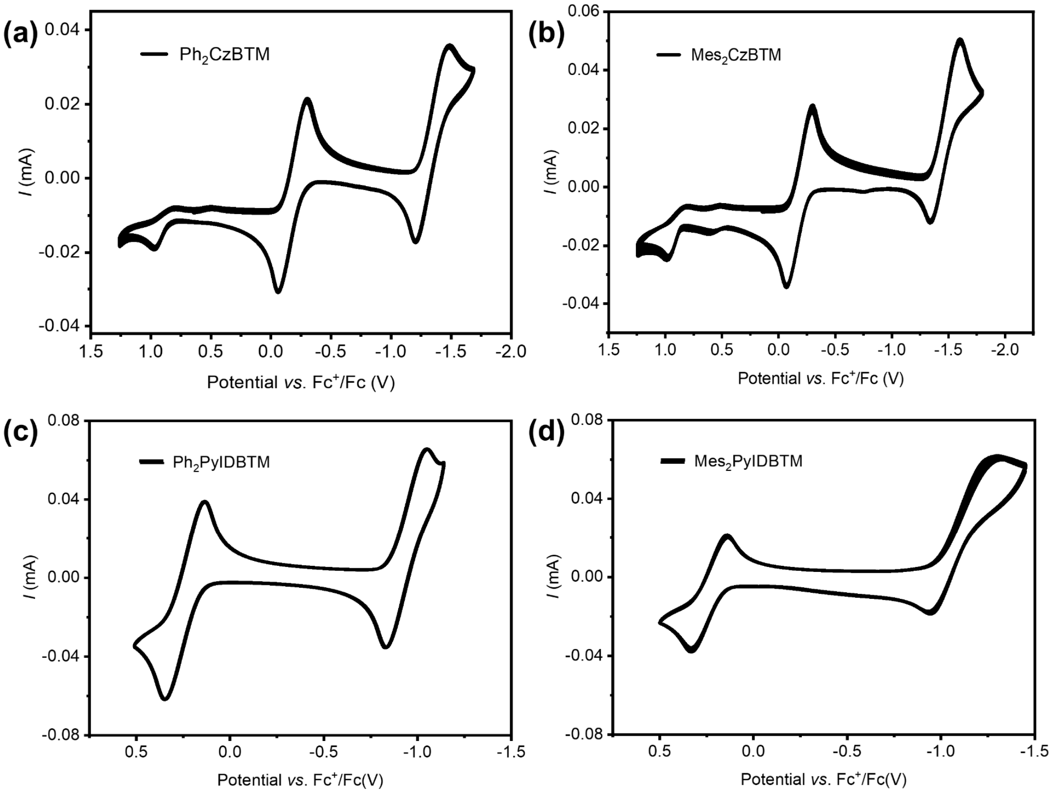

Figure S17. Multi-cycle CV measurements (20 cycles) of radicals: (a) **Ph2CzBTM**; (b) **Mes2CzBTM**; (c) **Ph2PyIDBTM**; (d) **Mes2PyIDBTM**.

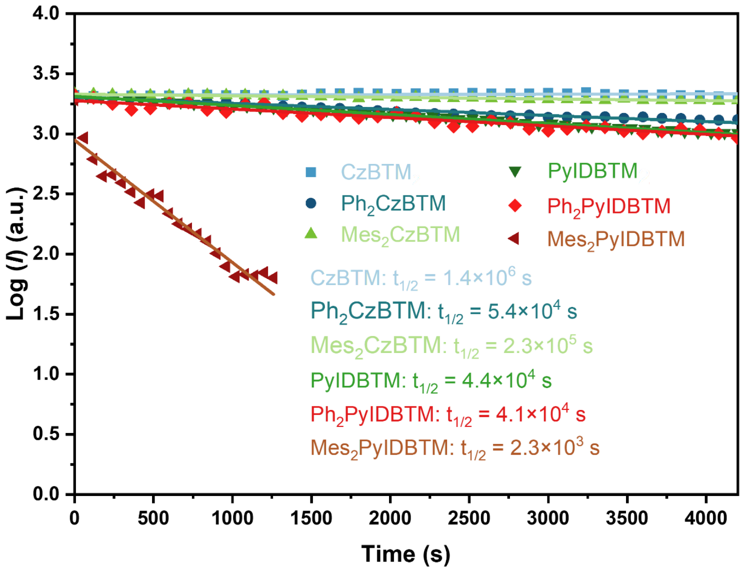

Figure S18. Photostability of radicals under continuous irradiation from xenon lamp in cyclohexane at room temperature.

Appendix (Calculation data).

CzBTM (UB3LYP/6-31G(d,p))

Center Number	Atomic Number	Atomic Type	Coordinates (Angstroms) X	Y	Z
1	6	0	-0.013509	0.167200	0.443877
2	6	0	1.113162	2.219276	-0.468576
3	6	0	-1.243988	2.139438	-0.509676
4	6	0	2.473490	1.978034	-0.492730
5	6	0	0.615448	3.541575	-0.254427
6	6	0	-2.583210	1.806715	-0.580786
7	6	0	-0.844632	3.492127	-0.279904
8	6	0	3.351724	3.055585	-0.303222
9	1	0	2.866841	0.965164	-0.656682
10	6	0	1.489765	4.599292	-0.068244
11	6	0	-3.538373	2.822129	-0.422980
12	1	0	-2.901195	0.769740	-0.757055
13	6	0	-1.794430	4.488090	-0.125506
14	6	0	2.867826	4.343669	-0.094667
15	1	0	4.436694	2.874990	-0.320512
16	1	0	1.114721	5.619016	0.097090
17	6	0	-3.150281	4.139803	-0.199512
18	1	0	-4.607387	2.568522	-0.477513
19	1	0	-1.495273	5.530699	0.051421
20	1	0	3.572598	5.175491	0.051836
21	1	0	-3.914380	4.921866	-0.078597
22	6	0	-1.237677	-0.750669	0.269151
23	6	0	-2.131516	-0.929895	1.325277
24	6	0	-1.452930	-1.402745	-0.944937
25	6	0	-3.239963	-1.761559	1.167481
26	6	0	-2.562203	-2.233922	-1.103266
27	6	0	-3.455612	-2.413526	-0.047283
28	1	0	-3.944199	-1.903370	2.000090
29	1	0	-2.731888	-2.747603	-2.060769
30	6	0	1.275309	-0.665516	0.312980
31	6	0	2.036877	-0.954828	1.445580
32	6	0	1.681227	-1.130596	-0.937812
33	6	0	3.204445	-1.708484	1.327226
34	6	0	2.848601	-1.885330	-1.056233

35	6	0	3.610290	-2.174210	0.075984
36	1	0	3.805108	-1.936032	2.219820
37	1	0	3.168184	-2.252239	-2.042498
38	7	0	-0.031578	1.259240	-0.641813
39	17	0	-1.858790	-0.107844	2.857417
40	17	0	-0.325664	-1.176606	-2.277503
41	17	0	-4.854536	-3.462859	-0.246062
42	17	0	1.524911	-0.366711	3.023430
43	17	0	0.720544	-0.766017	-2.366717
44	17	0	5.083664	-3.125306	-0.072960

Ph_2CzBTM (UB3LYP/6-31G(d,p))

Center Number	Atomic Number	Atomic Type	Coordinates (Angstroms) X	Y	Z
1	6	0	0.022262	0.847695	0.497185
2	6	0	1.225778	-0.097742	0.326025
3	6	0	1.109505	-1.238172	-0.468613
4	6	0	2.432652	0.186835	0.965464
5	6	0	2.200024	-2.094432	-0.623719
6	6	0	3.522808	-0.669425	0.810823
7	6	0	3.406301	-1.810607	0.016250
8	1	0	2.107802	-2.993851	-1.249644
9	1	0	4.474033	-0.445815	1.315270
10	6	0	-1.284687	0.042972	0.371071
11	6	0	-1.776179	-0.665576	1.467403
12	6	0	-1.977395	0.023143	-0.839785
13	6	0	-2.960346	-1.394364	1.352995
14	6	0	-3.161033	-0.705833	-0.954377
15	6	0	-3.652442	-1.415090	0.142295
16	1	0	-3.347158	-1.953488	2.217285
17	1	0	-3.706930	-0.722277	-1.908852
18	6	0	-4.959089	-2.220220	0.015649
19	6	0	-4.920314	-3.553035	-0.393799
20	6	0	-6.181664	-1.616504	0.311028
21	6	0	-6.104170	-4.282321	-0.508248
22	1	0	-3.956724	-4.029035	-0.626308
23	6	0	-7.365318	-2.345413	0.196173
24	1	0	-6.211893	-0.565742	0.634233

25	6	0	-7.326540	-3.678648	-0.213945
26	1	0	-6.073330	-5.332842	-0.831815
27	1	0	-8.329225	-1.869669	0.428222
28	1	0	-8.259764	-4.253230	-0.304737
29	6	0	4.609829	-2.756162	-0.154184
30	6	0	5.432941	-2.642680	-1.274522
31	6	0	4.877121	-3.726013	0.812435
32	6	0	6.523353	-3.499259	-1.428614
33	1	0	5.222528	-1.878076	-2.036264
34	6	0	5.966976	-4.582733	0.658212
35	1	0	4.228201	-3.814986	1.695872
36	6	0	6.790123	-4.469514	-0.462871
37	1	0	7.171636	-3.410040	-2.312362
38	1	0	6.177547	-5.347783	1.419562
39	1	0	7.649212	-5.145127	-0.584571
40	6	0	1.348462	2.713728	-0.538230
41	6	0	-0.988054	2.983161	-0.358451
42	6	0	2.650041	2.274229	-0.686577
43	6	0	1.073397	4.094351	-0.292725
44	6	0	-2.362389	2.852188	-0.300788
45	6	0	-0.373902	4.261251	-0.181383
46	6	0	3.691372	3.209579	-0.591323
47	1	0	2.872666	1.214894	-0.875678
48	6	0	2.107636	5.010675	-0.200203
49	6	0	-3.138386	3.997064	-0.065435
50	1	0	-2.844866	1.874150	-0.435549
51	6	0	-1.147803	5.386108	0.050285
52	6	0	3.424343	4.554340	-0.352309
53	1	0	4.731412	2.870723	-0.707801
54	1	0	1.904177	6.074079	-0.011508
55	6	0	-2.541078	5.242207	0.106781
56	1	0	-4.233452	3.904361	-0.017314
57	1	0	-0.682972	6.372498	0.187397
58	1	0	4.254626	5.272427	-0.281067
59	1	0	-3.166806	6.128186	0.290078
60	7	0	0.063573	1.933926	-0.593685
61	17	0	-0.902683	-0.640208	2.995134
62	17	0	-1.357192	0.917878	-2.222680
63	17	0	2.579095	1.626298	1.967512
64	17	0	-0.412781	-1.597001	-1.275760

Mes_2CzBTM (UB3LYP/6-31G(d,p))

Center Number	Atomic Number	Atomic Type	Coordinates (Angstroms) X	Y	Z
1	6	0	-0.016290	1.287686	0.505618
2	6	0	1.218911	0.378200	0.368819
3	6	0	1.151537	-0.777455	-0.409301
4	6	0	2.405570	0.710579	1.022864
5	6	0	2.270753	-1.601140	-0.533279
6	6	0	3.524416	-0.113110	0.899346
7	6	0	3.456832	-1.269523	0.121298
8	1	0	2.217085	-2.512571	-1.146185
9	1	0	4.459722	0.148184	1.415315
10	6	0	-1.295132	0.440240	0.371457
11	6	0	-1.782197	-0.266480	1.470940
12	6	0	-1.966819	0.379868	-0.849854
13	6	0	-2.940905	-1.033979	1.349235
14	6	0	-3.124991	-0.387805	-0.971732
15	6	0	-3.611956	-1.095226	0.128103
16	1	0	-3.324211	-1.591642	2.216028
17	1	0	-3.654293	-0.436202	-1.934433
18	6	0	1.299607	3.129749	-0.584421
19	6	0	-1.030878	3.417168	-0.359817
20	6	0	2.594994	2.679570	-0.753495
21	6	0	1.038451	4.515565	-0.353411
22	6	0	-2.404498	3.296115	-0.271547
23	6	0	-0.405113	4.693605	-0.214304
24	6	0	3.643962	3.609308	-0.693688
25	1	0	2.806929	1.616164	-0.931715
26	6	0	2.080152	5.426287	-0.295910
27	6	0	-3.168163	4.449373	-0.036776
28	1	0	-2.895844	2.319435	-0.381900
29	6	0	-1.166886	5.826779	0.017030
30	6	0	3.390478	4.959120	-0.468812
31	1	0	4.679175	3.261946	-0.826909
32	1	0	1.887395	6.493629	-0.118555
33	6	0	-2.559546	5.692909	0.104719
34	1	0	-4.262540	4.364604	0.035562
35	1	0	-0.693080	6.811961	0.129980
36	1	0	4.226577	5.672667	-0.425427
37	1	0	-3.175700	6.585557	0.288091
38	7	0	0.008954	2.357722	-0.601624

39	17	0	-0.345246	-1.196588	-1.234872
40	17	0	2.490306	2.169235	2.004058
41	17	0	-0.935233	-0.189976	3.011849
42	17	0	-1.352233	1.272305	-2.236735
43	6	0	4.692035	-2.179114	-0.014773
44	6	0	5.463247	-2.139120	-1.176316
45	6	0	5.039941	-3.042831	1.024152
46	6	0	6.582367	-2.963086	-1.299286
47	6	0	6.158506	-3.866971	0.901089
48	6	0	6.929734	-3.827307	-0.261172
49	1	0	7.189725	-2.931875	-2.215465
50	1	0	6.432621	-4.548417	1.719495
51	6	0	-4.890474	-1.943076	-0.006579
52	6	0	-4.815846	-3.243375	-0.505781
53	6	0	-6.123406	-1.410627	0.371295
54	6	0	-5.974213	-4.011386	-0.627500
55	6	0	-7.281600	-2.178229	0.249189
56	6	0	-7.206978	-3.478901	-0.250710
57	1	0	-5.915145	-5.036211	-1.021818
58	1	0	-8.253696	-1.758618	0.546265
59	6	0	-3.454884	-3.831372	-0.922485
60	1	0	-3.077806	-3.293948	-1.767403
61	1	0	-2.765302	-3.746068	-0.108790
62	1	0	-3.575943	-4.862645	-1.180790
63	6	0	-8.485781	-4.326250	-0.385848
64	1	0	-8.641657	-4.575127	-1.414762
65	1	0	-8.380727	-5.224270	0.186361
66	1	0	-9.323477	-3.768097	-0.023040
67	6	0	-6.205296	0.024843	0.922947
68	1	0	-5.581694	0.110536	1.788209
69	1	0	-5.873655	0.713984	0.174617
70	1	0	-7.217437	0.247379	1.189307
71	6	0	5.079575	-1.185423	-2.322988
72	1	0	4.113414	-1.450187	-2.698931
73	1	0	5.057013	-0.179822	-1.958080
74	1	0	5.801721	-1.263625	-3.108666
75	6	0	4.188533	-3.086269	2.306656
76	1	0	4.226902	-2.133786	2.792664
77	1	0	3.174745	-3.317233	2.054093
78	1	0	4.572392	-3.837971	2.964302
79	6	0	8.164510	-4.737491	-0.397165
80	1	0	9.021458	-4.238445	0.004697

81	1	0	7.997474	-5.647795	0.139813
82	1	0	8.332526	-4.958632	-1.430493

PyIDBTM (UB3LYP/6-31G(d,p))

Center Number	Atomic Number	Atomic Type	Coordinates (Angstroms) X	Y	Z
1	6	0	0.016674	0.166373	0.443695
2	6	0	1.237284	2.144367	-0.510569
3	6	0	-1.120195	2.213068	-0.468243
4	6	0	2.578030	1.818019	-0.582480
5	6	0	0.831647	3.495158	-0.280619
6	6	0	-2.479375	1.965355	-0.491566
7	6	0	-0.628637	3.537719	-0.254419
8	1	0	2.900822	0.782563	-0.758964
9	6	0	1.776824	4.495574	-0.126772
10	6	0	-3.362572	3.038713	-0.301373
11	1	0	-2.868016	0.950620	-0.655191
12	6	0	-1.507828	4.591329	-0.067798
13	6	0	3.134271	4.153759	-0.201559
14	1	0	1.472771	5.536716	0.050437
15	6	0	-2.884675	4.329132	-0.093407
16	1	0	-4.446683	2.852936	-0.317776
17	1	0	-1.137588	5.612872	0.097143
18	1	0	3.894728	4.939436	-0.081094
19	1	0	-3.593294	5.157613	0.053457
20	6	0	1.245102	-0.745672	0.268374
21	6	0	2.140073	-0.920973	1.324200
22	6	0	1.462963	-1.396571	-0.945881
23	6	0	3.252991	-1.746473	1.165497
24	6	0	2.575639	-2.223135	-1.104474
25	6	0	3.470694	-2.398068	-0.049100
26	1	0	3.958799	-1.884201	1.997460
27	1	0	2.746934	-2.736537	-2.061840
28	6	0	-1.268237	-0.672466	0.313487
29	6	0	-2.028086	-0.964967	1.446423
30	6	0	-1.672453	-1.139716	-0.937046
31	6	0	-3.191512	-1.725124	1.328855
32	6	0	-2.836738	-1.899286	-1.054962

33	6	0	-3.596201	-2.192157	0.077726
34	1	0	-3.790148	-1.956247	2.221891
35	1	0	-3.155420	-2.267211	-2.041140
36	17	0	-1.516951	-0.376191	3.024297
37	17	0	-0.714244	-0.770633	-2.366456
38	17	0	-5.064448	-3.151298	-0.070254
39	17	0	1.865390	-0.098470	2.855747
40	17	0	0.333961	-1.175832	-2.277883
41	17	0	4.875157	-3.439769	-0.248963
42	7	0	0.028994	1.258446	-0.642043
43	7	0	3.528479	2.837979	-0.425474

Ph_2PyIDBTM (UB3LYP/6-31G(d,p))

Center Number	Atomic Number	Atomic Type	Coordinates (Angstroms) X	Y	Z
1	6	0	0.009743	0.851912	0.484763
2	6	0	1.191673	2.843453	-0.489802
3	6	0	-1.167121	2.853477	-0.475335
4	6	0	2.540906	2.549792	-0.540875
5	6	0	0.749742	4.187325	-0.287917
6	6	0	-2.519321	2.571254	-0.509726
7	6	0	-0.711356	4.193540	-0.278974
8	1	0	2.891517	1.519933	-0.695711
9	6	0	1.667759	5.213704	-0.140768
10	6	0	-3.431230	3.625117	-0.348178
11	1	0	-2.880540	1.544410	-0.660109
12	6	0	-1.618726	5.227706	-0.120610
13	6	0	3.034107	4.904956	-0.194261
14	1	0	1.335730	6.249731	0.014906
15	6	0	-2.988197	4.930521	-0.157236
16	1	0	-4.510095	3.411847	-0.373615
17	1	0	-1.276086	6.260913	0.030763
18	1	0	3.773230	5.711474	-0.078849
19	1	0	-3.718969	5.743279	-0.032786
20	7	0	0.007521	1.925271	-0.619544
21	7	0	3.463667	3.595893	-0.390847
22	6	0	1.262547	-0.031769	0.339189
23	6	0	2.150360	-0.164623	1.407182
24	6	0	1.509642	-0.699413	-0.860287
25	6	0	3.285339	-0.964424	1.275413

26	6	0	2.644406	-1.500281	-0.991929
27	6	0	3.532298	-1.632773	0.075606
28	1	0	3.985496	-1.068689	2.116962
29	1	0	2.838756	-2.026897	-1.937644
30	6	0	-1.252193	-0.021114	0.354607
31	6	0	-2.022232	-0.306602	1.482441
32	6	0	-1.625417	-0.526344	-0.890783
33	6	0	-3.164846	-1.097706	1.364932
34	6	0	-2.768898	-1.316890	-1.008665
35	6	0	-3.538538	-1.602743	0.118935
36	1	0	-3.771501	-1.323292	2.253964
37	1	0	-3.063152	-1.714764	-1.990798
38	6	0	4.785559	-2.515877	-0.069534
39	6	0	4.828656	-3.770971	0.538210
40	6	0	5.877537	-2.061063	-0.808638
41	6	0	5.963780	-4.570721	0.407389
42	1	0	3.967898	-4.128928	1.121480
43	6	0	7.012698	-2.861346	-0.940418
44	1	0	5.843669	-1.071953	-1.287850
45	6	0	7.056042	-4.115952	-0.332452
46	1	0	5.998069	-5.559750	0.886914
47	1	0	7.873364	-2.502654	-1.523572
48	1	0	7.950915	-4.746727	-0.435510
49	6	0	-4.800281	-2.476158	-0.010485
50	6	0	-4.790575	-3.789701	0.459602
51	6	0	-5.952808	-1.954056	-0.597546
52	6	0	-5.932971	-4.581108	0.342003
53	1	0	-3.881545	-4.201267	0.921683
54	6	0	-7.095946	-2.745256	-0.714365
55	1	0	-5.960688	-0.918742	-0.967940
56	6	0	-7.086167	-4.058645	-0.244842
57	1	0	-5.925198	-5.616667	0.711940
58	1	0	-8.004647	-2.333201	-1.176909
59	1	0	-7.986803	-4.682811	-0.337403
60	17	0	-1.550184	0.330121	3.053859
61	17	0	-0.654352	-0.166122	-2.313763
62	17	0	1.838764	0.679003	2.920062
63	17	0	0.389681	-0.532222	-2.207631

Mes_2PyIDBTM (UB3LYP/6-31G(d,p))

Center Atomic Atomic Coordinates (Angstroms)

Number	Number	Type	X	Y	Z
1	6	0	-0.001790	1.272337	0.531438
2	6	0	1.175604	3.290403	-0.392750
3	6	0	-1.183236	3.284502	-0.400462
4	6	0	2.527225	3.006752	-0.436757
5	6	0	0.722657	4.627097	-0.168831
6	6	0	-2.533100	2.994049	-0.453187
7	6	0	-0.738469	4.623448	-0.173625
8	1	0	2.886279	1.982476	-0.608365
9	6	0	1.632242	5.656456	0.007058
10	6	0	-3.453641	4.038472	-0.279722
11	1	0	-2.885892	1.967980	-0.627037
12	6	0	-1.654307	5.648271	-0.003685
13	6	0	3.001103	5.357882	-0.039500
14	1	0	1.291719	6.687006	0.179801
15	6	0	-3.021319	5.342788	-0.059101
16	1	0	-4.530742	3.818593	-0.319554
17	1	0	-1.320146	6.680559	0.171102
18	1	0	3.733601	6.166864	0.098662
19	1	0	-3.758744	6.148086	0.074279
20	6	0	-1.256474	0.393682	0.372273
21	6	0	-1.509333	-0.248294	-0.839947
22	6	0	-2.139989	0.239406	1.440922
23	6	0	-2.645954	-1.044445	-0.983852
24	6	0	-3.276605	-0.556157	1.296975
25	6	0	-3.529814	-1.197985	0.084061
26	1	0	-2.845422	-1.549814	-1.939914
27	1	0	-3.973610	-0.677413	2.138864
28	6	0	1.258314	0.400009	0.380506
29	6	0	1.631863	-0.074341	-0.876873
30	6	0	2.026584	0.084650	1.501541
31	6	0	2.773616	-0.864422	-1.013415
32	6	0	3.167812	-0.705630	1.365179
33	6	0	3.541211	-1.180593	0.107324
34	1	0	3.067417	-1.238873	-2.004732
35	1	0	3.773222	-0.955001	2.248688
36	6	0	-4.784924	-2.076059	-0.074956
37	6	0	-5.887570	-1.595497	-0.781199
38	6	0	-4.818789	-3.352553	0.487028
39	6	0	-7.024446	-2.391243	-0.925331
40	6	0	-5.955536	-4.147981	0.343376

41	6	0	-7.058834	-3.666990	-0.362739
42	1	0	-7.893877	-2.011790	-1.481521
43	1	0	-5.982860	-5.153917	0.786822
44	6	0	4.801025	-2.053419	-0.043154
45	6	0	5.959550	-1.515440	-0.603462
46	6	0	4.783621	-3.382236	0.381527
47	6	0	7.100833	-2.306290	-0.739485
48	6	0	5.924445	-4.173092	0.245134
49	6	0	7.083294	-3.635003	-0.315887
50	1	0	8.013795	-1.882083	-1.181966
51	1	0	5.910872	-5.220744	0.579107
52	6	0	5.979015	-0.048526	-1.071846
53	1	0	5.279568	0.079916	-1.871332
54	1	0	5.709816	0.589846	-0.256424
55	1	0	6.961184	0.203878	-1.413219
56	6	0	3.504496	-3.975661	1.000627
57	1	0	3.269429	-3.452303	1.903811
58	1	0	2.694043	-3.875658	0.309205
59	1	0	3.661272	-5.011337	1.219021
60	6	0	8.342734	-4.508296	-0.466792
61	1	0	8.365879	-4.934316	-1.448052
62	1	0	9.214437	-3.905655	-0.318949
63	1	0	8.322951	-5.291684	0.261776
64	6	0	-3.600954	-3.882983	1.266209
65	1	0	-2.739104	-3.871082	0.632194
66	1	0	-3.424895	-3.261356	2.119135
67	1	0	-3.792704	-4.885054	1.588674
68	6	0	-5.850120	-0.186655	-1.401973
69	1	0	-5.661689	0.535890	-0.635603
70	1	0	-5.071918	-0.140412	-2.134887
71	1	0	-6.790733	0.023429	-1.866745
72	6	0	-8.314018	-4.545088	-0.521026
73	1	0	-9.186211	-3.925318	-0.512959
74	1	0	-8.262791	-5.076697	-1.448209
75	1	0	-8.365161	-5.243358	0.288112
76	17	0	-0.394556	-0.054177	-2.187979
77	17	0	-1.820173	1.049119	2.970524
78	17	0	1.555216	0.683958	3.087808
79	17	0	0.663050	0.323863	-2.291241
80	7	0	-0.000999	2.367112	-0.551644
81	7	0	3.441393	4.055803	-0.257560

Excited states calculated by TD-DFT calculations

CzBTM (UB3LYP/6-31G(d))

Excited State 1: 2.055-A 2.1924 eV 565.52 nm f=0.0891 <S**2>=0.806

134B ->136B 0.98719

This state for optimization and/or second-order correction.

Total Energy, E(TD-HF/TD-DFT) = -3775.72772659

Copying the excited state density for this state as the 1-particle RhoCI density.

Excited State 2: 2.071-A 2.2585 eV 548.98 nm f=0.0000 <S**2>=0.823

135B ->136B 0.98767

Excited State 3: 2.187-A 2.5895 eV 478.80 nm f=0.0467 <S**2>=0.945

134A ->137A 0.14559

136A ->137A 0.90060

130B ->136B 0.26309

133B ->136B 0.15720

Excited State 4: 2.100-A 2.9800 eV 416.06 nm f=0.0015 <S**2>=0.853

134A ->138A 0.11091

136A ->138A 0.92880

131B ->136B 0.30741

Excited State 5: 2.143-A 3.0387 eV 408.02 nm f=0.0000 <S**2>=0.899

136A ->139A 0.91626

132B ->136B 0.32992

Excited State 6: 2.664-A 3.1134 eV 398.23 nm f=0.0210 <S**2>=1.524

134A ->144A -0.14389

135A ->139A -0.17837

135A ->140A 0.26707

136A ->137A -0.19480

136A ->144A 0.18167

133B ->136B 0.73886

134B ->144B 0.16013

135B ->136B -0.12034

135B ->139B 0.24483

135B ->140B -0.26328

Excited State 7: 2.461-A 3.2086 eV 386.41 nm f=0.0181 <S**2>=1.264

130A ->137A -0.21696

131A ->139A 0.12340

132A ->138A 0.13650
133A ->137A -0.10621
134A ->141A 0.14139
136A ->139A 0.10182
136A ->141A 0.46857
136A ->143A -0.22585
129B ->136B 0.59017
130B ->137B 0.18811
131B ->138B 0.14654
132B ->136B -0.32651
132B ->139B 0.11194

Excited State 8: 2.361-A 3.2301 eV 383.84 nm f=0.0027 <S**2>=1.144
134A ->139A 0.12887
136A ->139A -0.22005
136A ->140A 0.80644
129B ->136B 0.13121
132B ->136B 0.41926
134B ->139B -0.14993
134B ->140B 0.13422

Excited State 9: 2.815-A 3.2415 eV 382.49 nm f=0.0706 <S**2>=1.731
134A ->137A 0.12435
134A ->144A 0.10572
135A ->139A 0.23115
135A ->140A -0.34068
136A ->137A -0.28693
136A ->138A -0.14117
136A ->144A -0.12819
130B ->136B 0.44331
131B ->136B 0.31295
133B ->136B 0.33668
134B ->144B -0.12353
135B ->139B -0.27469
135B ->140B 0.29115

Excited State 10: 2.210-A 3.3224 eV 373.18 nm f=0.0127 <S**2>=0.971
135A ->139A -0.10545
135A ->140A 0.14987
136A ->138A -0.29159
130B ->136B -0.17877
131B ->136B 0.86180

133B ->136B -0.22052
135B ->140B -0.10060

Excited State 11: 2.184-A 3.3561 eV 369.43 nm f=0.0038 <S**2>=0.943
136A ->139A -0.25161
136A ->140A -0.47778
136A ->141A 0.21571
129B ->136B 0.18300
132B ->136B 0.75179
134B ->139B 0.10151

Excited State 12: 2.463-A 3.3763 eV 367.22 nm f=0.0583 <S**2>=1.266
134A ->137A 0.14916
135A ->139A -0.19101
135A ->140A 0.26694
136A ->137A -0.19346
130B ->136B 0.70422
133B ->136B -0.46222
135B ->139B 0.15989
135B ->140B -0.16382

Excited State 13: 2.428-A 3.6753 eV 337.34 nm f=0.0692 <S**2>=1.224
130A ->137A 0.12843
132A ->138A -0.11043
133A ->144A 0.10282
136A ->140A 0.10997
136A ->141A 0.79535
136A ->143A 0.17146
136A ->145A 0.13162
129B ->136B -0.32222
131B ->138B -0.12509
132B ->139B -0.10323

Excited State 14: 3.083-A 3.7489 eV 330.72 nm f=0.0172 <S**2>=2.126
131A ->138A -0.28664
132A ->139A -0.23130
132A ->140A -0.12408
134A ->137A -0.33760
136A ->137A 0.11039
136A ->142A -0.36823
129B ->137B -0.14534
130B ->136B 0.36899

131B ->136B 0.10519
131B ->139B -0.19820
131B ->140B -0.14908
132B ->138B -0.26598
133B ->136B 0.11080
134B ->137B 0.41270

Excited State 15: 2.309-A 3.7610 eV 329.66 nm f=0.0014 <S**2>=1.083
131A ->138A -0.11611
134A ->137A -0.19003
136A ->142A 0.90851
130B ->136B 0.12277
132B ->138B -0.11215
134B ->137B 0.16057

Excited State 16: 3.038-A 3.8410 eV 322.80 nm f=0.0133 <S**2>=2.058
130A ->137A 0.17029
130A ->138A -0.13331
131A ->139A -0.31573
131A ->140A -0.16589
132A ->138A -0.34940
136A ->145A -0.10555
129B ->136B 0.54895
130B ->137B -0.20192
131B ->138B -0.32160
132B ->139B -0.25664
132B ->140B -0.18970
133B ->137B -0.10971
134B ->139B -0.11769

Excited State 17: 2.626-A 3.9034 eV 317.63 nm f=0.0121 <S**2>=1.474
133A ->144A -0.15032
134A ->139A 0.12583
134A ->140A -0.14509
134A ->141A 0.13280
136A ->140A -0.14495
136A ->141A 0.13516
136A ->143A 0.74071
136A ->145A -0.11334
128B ->136B -0.12270
131B ->138B 0.11369
133B ->144B 0.13696

134B ->139B -0.27466
134B ->140B 0.20493
134B ->141B -0.11552

Excited State 18: 2.447-A 3.9703 eV 312.28 nm f=0.0205 <S**2>=1.247
134A ->139A -0.10775
134A ->140A 0.13190
136A ->140A 0.10085
136A ->141A -0.14398
136A ->143A 0.48243
128B ->136B 0.58608
129B ->136B 0.27837
134B ->139B 0.34361
134B ->140B -0.25783

Excited State 19: 2.742-A 3.9955 eV 310.31 nm f=0.0067 <S**2>=1.630
134A ->144A -0.18746
136A ->144A 0.78536
133B ->136B -0.12234
133B ->139B -0.11048
133B ->140B 0.11558
134B ->144B 0.26340
135B ->139B -0.30094
135B ->140B 0.24870

Excited State 20: 2.876-A 4.0042 eV 309.63 nm f=0.0001 <S**2>=1.817
134A ->140A 0.10922
135A ->137A 0.89101
135A ->144A -0.10116
136A ->140A 0.10076
136A ->143A 0.13105
128B ->136B -0.27338
134B ->139B 0.17632
134B ->140B -0.13259

Excited State 21: 2.451-A 4.0255 eV 308.00 nm f=0.0019 <S**2>=1.252
134A ->139A 0.16815
134A ->140A -0.20980
135A ->137A 0.44120
135A ->144A 0.14587
136A ->140A -0.18820
136A ->141A 0.10131

136A ->143A -0.21675
128B ->136B 0.67382
134B ->139B -0.24910
134B ->140B 0.19153
135B ->137B 0.12310

Excited State 22: 3.167-A 4.0621 eV 305.22 nm f=0.0139 <S**2>=2.257
131A ->138A 0.30477
132A ->139A 0.24602
132A ->140A 0.12476
134A ->137A -0.10201
131B ->139B 0.20521
131B ->140B 0.14805
132B ->138B 0.27716
134B ->137B 0.79102

Excited State 23: 2.814-A 4.1491 eV 298.82 nm f=0.0002 <S**2>=1.730
128B ->136B -0.15117
135B ->137B 0.98412

Excited State 24: 2.473-A 4.1827 eV 296.42 nm f=0.0825 <S**2>=1.279
131A ->138A -0.15705
132A ->139A -0.11964
134A ->137A 0.82128
126B ->136B 0.10380
131B ->139B -0.14758
131B ->140B -0.10145
132B ->138B -0.19833
134B ->137B 0.37449
134B ->138B -0.11591

Excited State 25: 3.336-A 4.2475 eV 291.90 nm f=0.0023 <S**2>=2.533
131A ->137A -0.13938
133A ->139A 0.10679
134A ->137A 0.10617
134A ->138A -0.38721
136A ->138A 0.10816
129B ->138B -0.14301
133B ->139B -0.11954
134B ->138B 0.82098

Excited State 26: 3.103-A 4.2628 eV 290.85 nm f=0.0090 <S**2>=2.157

128A ->140A 0.10241
133A ->144A -0.18374
134A ->141A 0.15153
134A ->145A 0.14628
135A ->138A 0.27849
135A ->144A -0.22038
135A ->146A -0.17838
136A ->141A 0.10897
136A ->143A -0.12739
136A ->145A -0.27002
127B ->136B -0.13582
128B ->136B 0.14058
128B ->139B -0.11612
128B ->140B 0.13206
129B ->136B -0.17153
133B ->144B 0.20889
134B ->139B 0.43014
134B ->140B -0.14962
134B ->141B -0.25119
134B ->143B 0.21671
134B ->145B 0.17070
135B ->138B -0.12804
135B ->144B 0.10401
135B ->146B -0.16128

Excited State 27: 3.376-A 4.3471 eV 285.21 nm f=0.0011 <S**2>=2.599
130A ->138A 0.17638
132A ->137A -0.23690
133A ->138A 0.12360
134A ->139A -0.31176
134A ->140A -0.26990
135A ->144A 0.13378
136A ->139A 0.11157
129B ->136B 0.10489
129B ->139B -0.12985
130B ->138B -0.19805
131B ->137B -0.18123
133B ->138B -0.13707
134B ->139B 0.37055
134B ->140B 0.58318

Excited State 28: 2.821-A 4.3618 eV 284.25 nm f=0.0018 <S**2>=1.740

135A ->138A 0.95140
136A ->145A 0.10401
134B ->139B -0.13903

Excited State 29: 2.582-A 4.4221 eV 280.37 nm f=0.0208 <S**2>=1.416
133A ->139A 0.18450
133A ->140A -0.14750
134A ->138A -0.34965
135A ->139A 0.73346
135A ->140A 0.10832
136A ->144A -0.18578
133B ->139B -0.11670
134B ->138B -0.25263
134B ->144B 0.21634
135B ->139B 0.18814

Excited State 30: 2.854-A 4.4263 eV 280.11 nm f=0.0000 <S**2>=1.786
130A ->137A -0.12633
130B ->137B 0.11822
134B ->139B 0.13212
135B ->138B 0.92399

Ph_2CzBTM (UB3LYP/6-31G(d))

Excited State 1: 2.068-A 2.2051 eV 562.25 nm f=0.1018 <S**2>=0.819
159B ->160B 0.98442
This state for optimization and/or second-order correction.
Total Energy, E(TD-HF/TD-DFT) = -3318.67193087
Copying the excited state density for this state as the 1-particle RhoCI density.

Excited State 2: 2.082-A 2.3228 eV 533.78 nm f=0.0004 <S**2>=0.833
160A ->161A 0.10857
158B ->160B 0.97849

Excited State 3: 2.202-A 2.3914 eV 518.46 nm f=0.0894 <S**2>=0.963
158A ->161A 0.12418
160A ->161A 0.89582
157B ->160B 0.29498
158B ->160B -0.12382

Excited State 4: 2.461-A 2.8988 eV 427.70 nm f=0.0224 <S**2>=1.264
157A ->161A -0.23948

158A ->162A 0.15615
160A ->162A 0.61029
160A ->163A -0.10742
148B ->160B 0.16988
156B ->160B 0.58576
157B ->161B 0.22478

Excited State 5: 2.264-A 2.9591 eV 419.00 nm f=0.2970 <S**2>=1.031
158A ->161A 0.15832
160A ->161A -0.36971
160A ->164A 0.12174
150B ->160B 0.15700
155B ->160B -0.10170
156B ->161B 0.12108
157B ->160B 0.83866

Excited State 6: 2.142-A 3.0367 eV 408.29 nm f=0.0137 <S**2>=0.897
160A ->164A 0.74524
155B ->160B -0.60444
157B ->160B -0.12412

Excited State 7: 2.191-A 3.0504 eV 406.45 nm f=0.0008 <S**2>=0.950
158A ->165A -0.10006
160A ->162A 0.19481
160A ->163A 0.74814
160A ->165A -0.21368
154B ->160B 0.53509
156B ->160B -0.11469

Excited State 8: 2.273-A 3.1445 eV 394.29 nm f=0.0000 <S**2>=1.041
158A ->163A 0.13298
160A ->163A -0.56900
160A ->165A -0.42951
154B ->160B 0.63015
156B ->160B -0.13830
159B ->162B 0.14330

Excited State 9: 3.132-A 3.1717 eV 390.91 nm f=0.0162 <S**2>=2.202
149A ->174A -0.11879
154A ->163A -0.10508
158A ->168A 0.11172
158A ->169A -0.14321

159A ->163A 0.42677
159A ->165A 0.24297
160A ->168A -0.12913
160A ->169A 0.18068
149B ->173B 0.12233
153B ->160B -0.46239
153B ->162B -0.11488
158B ->160B 0.13472
158B ->162B 0.47059
158B ->165B 0.20045
159B ->168B -0.12866
159B ->169B 0.15392

Excited State 10: 2.113-A 3.2416 eV 382.47 nm f=0.0126 <S**2>=0.866
160A ->164A 0.62356
153B ->160B -0.10663
155B ->160B 0.75264

Excited State 11: 2.175-A 3.3258 eV 372.80 nm f=0.0104 <S**2>=0.932
158A ->163A -0.10042
160A ->163A -0.12643
160A ->165A 0.82083
154B ->160B 0.48678
159B ->162B -0.13019

Excited State 12: 2.568-A 3.3750 eV 367.36 nm f=0.0121 <S**2>=1.399
158A ->161A -0.10354
159A ->163A 0.34632
159A ->165A 0.19258
153B ->160B 0.81436
155B ->160B 0.12086
158B ->162B 0.27020
158B ->165B 0.11256

Excited State 13: 2.264-A 3.4037 eV 364.26 nm f=0.1217 <S**2>=1.032
157A ->161A 0.19613
158A ->162A -0.11180
160A ->162A 0.71642
160A ->163A -0.16547
160A ->170A 0.13549
154B ->160B -0.14374
156B ->160B -0.53613

Excited State 14: 3.206-A 3.5078 eV 353.46 nm f=0.0328 <S**2>=2.320
151A ->167A -0.13845
152A ->166A -0.13994
153A ->161A -0.19870
155A ->164A -0.13235
156A ->165A -0.10323
157A ->162A -0.24912
158A ->161A 0.42959
150B ->160B -0.17657
151B ->167B 0.13674
152B ->166B -0.13966
153B ->160B 0.20838
154B ->163B 0.11941
155B ->165B -0.10068
156B ->161B 0.25676
157B ->160B -0.32369
157B ->164B 0.23357
159B ->161B -0.42800

Excited State 15: 3.056-A 3.5800 eV 346.32 nm f=0.0079 <S**2>=2.086
151A ->166A 0.14779
152A ->167A 0.14646
153A ->162A 0.19669
155A ->165A -0.14470
156A ->164A -0.18790
157A ->161A 0.37342
158A ->162A -0.14788
148B ->160B 0.13448
151B ->166B -0.14289
152B ->167B 0.14022
154B ->160B 0.11855
154B ->165B -0.12452
155B ->163B 0.16178
156B ->160B 0.49412
156B ->164B -0.22481
157B ->161B -0.39857
159B ->164B 0.12833

Excited State 16: 3.113-A 3.6664 eV 338.17 nm f=0.0004 <S**2>=2.172
148A ->161A -0.11106
150A ->162A -0.11959

151A ->167A 0.15827
152A ->166A 0.16059
153A ->161A 0.16246
155A ->164A -0.21768
156A ->163A 0.11929
156A ->165A -0.16813
157A ->162A 0.10897
158A ->161A 0.20772
160A ->161A -0.10380
160A ->168A -0.21759
160A ->169A -0.12384
148B ->161B 0.16332
150B ->160B 0.47103
151B ->167B -0.15848
152B ->166B 0.16284
154B ->163B 0.20826
155B ->162B 0.10291
155B ->165B -0.17073
156B ->161B -0.12144
157B ->160B -0.16432
157B ->164B -0.11670
159B ->161B -0.24300

Excited State 17: 3.207-A 3.7353 eV 331.93 nm f=0.0014 <S**2>=2.321
150A ->161A -0.20888
151A ->166A 0.17564
152A ->167A 0.17340
153A ->162A 0.14804
155A ->163A -0.15796
155A ->165A 0.21899
156A ->164A 0.28607
157A ->168A 0.12977
160A ->170A 0.17470
160A ->172A -0.10066
148B ->160B 0.40393
150B ->161B 0.21400
151B ->166B -0.17698
152B ->167B 0.17251
154B ->162B -0.13339
154B ->165B 0.22658
155B ->163B -0.28162
156B ->160B -0.11437

156B ->164B -0.13845
157B ->168B -0.11230

Excited State 18: 2.248-A 3.7845 eV 327.61 nm f=0.0002 <S**2>=1.014
160A ->166A 0.14810
160A ->167A -0.22514
151B ->160B 0.48078
151B ->161B -0.14423
152B ->160B 0.78383

Excited State 19: 2.248-A 3.7846 eV 327.60 nm f=0.0004 <S**2>=1.013
160A ->166A 0.23603
160A ->167A 0.14120
151B ->160B 0.78115
152B ->160B -0.48252
152B ->161B -0.14434

Excited State 20: 2.874-A 3.8511 eV 321.94 nm f=0.0005 <S**2>=1.815
159A ->161A 0.99449

Excited State 21: 2.396-A 3.8702 eV 320.36 nm f=0.1561 <S**2>=1.185
155A ->164A -0.13993
156A ->165A -0.10634
158A ->161A 0.39943
150B ->160B 0.10326
154B ->163B 0.10894
156B ->161B 0.10345
157B ->160B -0.10296
159B ->161B 0.84275

Excited State 22: 3.145-A 3.8741 eV 320.04 nm f=0.0034 <S**2>=2.222
149A ->163A 0.13587
154A ->168A -0.12780
154A ->169A 0.16730
158A ->162A 0.11485
158A ->163A 0.32958
158A ->165A 0.16336
159A ->174A -0.11443
160A ->162A 0.12392
160A ->163A 0.16387
160A ->165A 0.19674
160A ->170A -0.20010

160A ->173A 0.10703
149B ->160B -0.12417
149B ->162B 0.14269
153B ->168B 0.11995
153B ->169B -0.14949
156B ->160B -0.10469
158B ->161B -0.11560
158B ->173B 0.13086
159B ->162B 0.57500
159B ->164B -0.11324
159B ->165B 0.20621
159B ->170B -0.10442

Excited State 23: 2.144-A 3.9342 eV 315.15 nm f=0.0031 <S**2>=0.899
160A ->166A 0.88714
160A ->168A 0.18140
160A ->171A 0.22055
151B ->160B -0.29811

Excited State 24: 2.794-A 3.9413 eV 314.58 nm f=0.0025 <S**2>=1.701
149B ->160B 0.13790
158B ->161B 0.97335

Excited State 25: 2.167-A 3.9436 eV 314.39 nm f=0.0002 <S**2>=0.924
160A ->165A 0.12402
160A ->167A 0.90623
160A ->170A 0.14616
152B ->160B 0.29266

Excited State 26: 2.658-A 3.9488 eV 313.98 nm f=0.0056 <S**2>=1.516
158A ->168A 0.12245
158A ->169A -0.14079
159A ->163A -0.10800
160A ->166A 0.10692
160A ->168A -0.50427
160A ->169A 0.62149
153B ->160B 0.15119
153B ->162B -0.13106
158B ->162B -0.35638
158B ->165B -0.12662
159B ->168B -0.15174
159B ->169B 0.18156

Excited State 27: 3.134-A 4.0142 eV 308.86 nm f=0.0628 <S**2>=2.206
153A ->161A 0.14004
155A ->164A 0.28723
156A ->163A -0.15405
156A ->165A 0.21307
158A ->161A 0.69447
160A ->168A -0.10330
150B ->160B -0.14252
154B ->163B -0.29675
155B ->162B -0.14829
155B ->165B 0.23285
156B ->161B -0.12088

Excited State 28: 2.293-A 4.0655 eV 304.97 nm f=0.0055 <S**2>=1.064
158A ->163A 0.17572
159A ->169A -0.11933
160A ->170A 0.13161
149B ->160B 0.89294
158B ->161B -0.16606
158B ->169B 0.10826
159B ->162B 0.10844

Excited State 29: 2.254-A 4.0830 eV 303.66 nm f=0.0148 <S**2>=1.020
155A ->164A 0.13186
160A ->166A -0.21346
160A ->168A 0.42673
160A ->169A 0.38978
160A ->171A 0.49028
150B ->160B 0.49436
154B ->163B -0.12753
155B ->165B 0.10448
157B ->160B -0.10331

Excited State 30: 2.395-A 4.1056 eV 301.99 nm f=0.0028 <S**2>=1.184
160A ->167A -0.16109
160A ->170A 0.76192
160A ->173A -0.12443
148B ->160B -0.21068
149B ->160B -0.26206
159B ->162B 0.34842
159B ->164B 0.12970

Mes2CzBTM (UB3LYP/6-31G(d))

```
Excited State   1:   2.057-A     2.2726 eV   545.55 nm   f=0.0900   <S**2>=0.808
     183B ->184B         0.98663
 This state for optimization and/or second-order correction.
 Total Energy, E(TD-HF/TD-DFT) =  -3554.58004198
 Copying the excited state density for this state as the 1-particle RhoCI density.

 Excited State   2:   2.082-A     2.3830 eV   520.29 nm   f=0.0000   <S**2>=0.834
     182B ->184B         0.98492

 Excited State   3:   2.209-A     2.6831 eV   462.10 nm   f=0.0619   <S**2>=0.970
     182A ->185A         0.15030
     184A ->185A         0.88892
     173B ->185B        -0.10648
     174B ->184B        -0.28626
     175B ->184B        -0.17165

 Excited State   4:   2.121-A     3.0639 eV   404.66 nm   f=0.0003   <S**2>=0.875
     182A ->187A         0.10565
     184A ->187A         0.75213
     176B ->184B        -0.60965
     178B ->184B        -0.11190

 Excited State   5:   2.220-A     3.0748 eV   403.23 nm   f=0.0016   <S**2>=0.982
     182A ->188A        -0.10600
     184A ->186A         0.80716
     184A ->188A        -0.18395
     177B ->184B        -0.49725
     183B ->186B         0.10868

 Excited State   6:   2.266-A     3.1602 eV   392.33 nm   f=0.0002   <S**2>=1.033
     182A ->186A        -0.12796
     184A ->186A         0.51085
     184A ->188A         0.44349
     177B ->184B         0.66660
     179B ->184B        -0.16093
     183B ->186B         0.14548

 Excited State   7:   3.010-A     3.1633 eV   391.95 nm   f=0.0169   <S**2>=2.015
     175A ->186A        -0.10574
     182A ->194A         0.16484
     183A ->186A        -0.40308
```

183A ->188A -0.21345
184A ->185A 0.14842
184A ->194A -0.20991
172B ->198B 0.11445
175B ->184B 0.53832
175B ->186B 0.11223
182B ->184B -0.14561
182B ->186B -0.43875
182B ->188B -0.17261
183B ->194B 0.19612

Excited State 8: 2.074-A 3.2190 eV 385.16 nm f=0.0000 <S**2>=0.826
180B ->184B 0.96419
181B ->184B 0.22008

Excited State 9: 2.092-A 3.2191 eV 385.15 nm f=0.0007 <S**2>=0.844
180B ->184B -0.22064
181B ->184B 0.96130

Excited State 10: 2.147-A 3.2724 eV 378.88 nm f=0.0175 <S**2>=0.902
184A ->187A 0.61213
174B ->184B 0.13459
175B ->184B 0.10031
176B ->184B 0.66486
178B ->184B 0.30233
182B ->186B 0.11079

Excited State 11: 2.488-A 3.2762 eV 378.44 nm f=0.0224 <S**2>=1.298
174A ->185A -0.20476
175A ->185A -0.10506
176A ->187A 0.15988
177A ->188A 0.13036
182A ->189A 0.15320
184A ->188A -0.37075
184A ->189A 0.45879
173B ->184B 0.58154
174B ->185B 0.17702
176B ->187B 0.16808
177B ->184B 0.13827
177B ->188B -0.13704

Excited State 12: 2.530-A 3.2917 eV 376.66 nm f=0.1256 <S**2>=1.350

182A ->185A -0.11706
183A ->186A 0.27682
183A ->188A 0.14493
184A ->185A 0.33572
184A ->187A -0.10109
174B ->184B 0.48577
175B ->184B 0.53987
176B ->184B -0.19056
178B ->184B -0.16786
182B ->186B 0.26497
182B ->188B 0.10331

Excited State 13: 2.092-A 3.3379 eV 371.44 nm f=0.0002 <S**2>=0.844
184A ->188A 0.32040
184A ->189A 0.10199
173B ->184B 0.10156
179B ->184B 0.92348

Excited State 14: 2.067-A 3.3471 eV 370.42 nm f=0.0014 <S**2>=0.818
184A ->187A -0.12654
176B ->184B -0.32780
178B ->184B 0.92796

Excited State 15: 2.194-A 3.3752 eV 367.34 nm f=0.0022 <S**2>=0.953
184A ->186A -0.12979
184A ->188A 0.67405
184A ->189A 0.22379
173B ->184B 0.20940
177B ->184B -0.50522
179B ->184B -0.32258
183B ->186B 0.10638

Excited State 16: 2.366-A 3.4640 eV 357.92 nm f=0.0636 <S**2>=1.149
182A ->185A -0.11303
183A ->186A -0.26695
183A ->188A -0.13005
184A ->185A 0.17795
174B ->184B 0.68037
175B ->184B -0.55843
182B ->186B -0.17573

Excited State 17: 3.469-A 3.6302 eV 341.54 nm f=0.0002 <S**2>=2.758

178A ->193A -0.30352
179A ->192A -0.29385
180A ->191A 0.39013
181A ->190A -0.39164
178B ->193B 0.30282
179B ->192B 0.30235
180B ->190B -0.38974
181B ->189B 0.39034

Excited State 18: 3.468-A 3.6305 eV 341.51 nm f=0.0000 <S**2>=2.757
178A ->192A -0.29432
179A ->193A -0.30372
180A ->190A 0.38973
181A ->191A -0.39086
178B ->192B 0.30241
179B ->193B 0.30254
180B ->189B -0.38951
181B ->190B 0.39053

Excited State 19: 2.688-A 3.7685 eV 329.00 nm f=0.0926 <S**2>=1.556
174A ->185A 0.10922
175A ->194A 0.15956
176A ->187A -0.11732
182A ->186A -0.17395
182A ->189A -0.11296
182A ->197A -0.10530
184A ->188A -0.12634
184A ->189A 0.69899
184A ->197A 0.16780
172B ->186B 0.10619
173B ->184B -0.29223
175B ->194B 0.14658
176B ->187B -0.14024
177B ->188B 0.11845
182B ->198B -0.10126
183B ->186B 0.24132
183B ->191B -0.13353

Excited State 20: 3.241-A 3.8159 eV 324.91 nm f=0.0136 <S**2>=2.377
176A ->186A 0.19389
176A ->188A -0.29021
177A ->187A -0.36086

182A ->185A 0.31361
184A ->185A -0.13501
173B ->185B -0.15323
174B ->184B 0.38631
175B ->184B 0.12394
176B ->186B 0.15511
176B ->188B -0.29585
177B ->187B 0.34540
183B ->185B -0.31704

Excited State 21: 3.105-A 3.8824 eV 319.35 nm f=0.0044 <S**2>=2.160
174A ->185A 0.13863
176A ->187A -0.38050
177A ->186A 0.19993
177A ->188A -0.30223
182A ->186A 0.13393
184A ->189A 0.10736
184A ->197A -0.10681
173B ->184B 0.48733
174B ->185B -0.15954
176B ->187B -0.35702
177B ->186B -0.15764
177B ->188B 0.30003
183B ->186B -0.20612

Excited State 22: 2.670-A 3.9559 eV 313.42 nm f=0.0077 <S**2>=1.532
182A ->194A -0.16423
183A ->186A -0.10917
184A ->192A -0.20570
184A ->194A 0.77566
174B ->184B -0.11873
175B ->184B 0.14109
175B ->186B -0.13383
182B ->186B -0.36479
182B ->188B -0.11249
183B ->194B -0.24013

Excited State 23: 2.848-A 3.9599 eV 313.10 nm f=0.0106 <S**2>=1.778
173A ->186A -0.10968
175A ->194A 0.11068
182A ->186A -0.30184
182A ->188A -0.15665

183A ->194A 0.10368
184A ->186A -0.18415
184A ->188A -0.13712
184A ->189A -0.38933
173B ->184B 0.37913
183B ->186B 0.60830
183B ->188B 0.16887

Excited State 24: 2.089-A 4.1054 eV 302.00 nm f=0.0054 <S**2>=0.841
184A ->191A -0.17041
184A ->192A -0.15010
184A ->195A 0.95398

Excited State 25: 2.273-A 4.1165 eV 301.19 nm f=0.0089 <S**2>=1.042
182A ->186A -0.15324
183A ->185A -0.12212
183A ->194A 0.16300
172B ->184B 0.91219
182B ->185B -0.10366
182B ->194B -0.15765

Excited State 26: 2.098-A 4.1431 eV 299.25 nm f=0.0002 <S**2>=0.850
184A ->190A 0.99218

Excited State 27: 2.117-A 4.1455 eV 299.08 nm f=0.0003 <S**2>=0.871
184A ->191A 0.97174
184A ->195A 0.16384

Excited State 28: 3.332-A 4.1702 eV 297.31 nm f=0.0028 <S**2>=2.525
176A ->186A 0.14583
176A ->188A -0.21085
177A ->187A -0.27125
182A ->185A -0.33129
184A ->191A -0.10378
176B ->186B 0.11668
176B ->188B -0.21345
177B ->187B 0.25773
183B ->185B 0.75652

Excited State 29: 2.815-A 4.2036 eV 294.95 nm f=0.0007 <S**2>=1.731
183A ->185A 0.95437
184A ->196A 0.17212

172B ->184B 0.14551

Excited State 30: 2.317-A 4.2363 eV 292.67 nm f=0.0006 <S**2>=1.092
183A ->185A -0.23834
183A ->194A -0.10256
184A ->196A 0.86143
184A ->197A 0.16032
171B ->184B -0.13257
183B ->186B -0.14452
183B ->191B -0.10359

PyIDBTM (UB3LYP/6-31G(d))

Excited State 1: 2.059-A 2.2747 eV 545.05 nm f=0.0874 <S**2>=0.810
135B ->136B 0.98590
This state for optimization and/or second-order correction.
Total Energy, E(TD-HF/TD-DFT) = -3791.75747652
Copying the excited state density for this state as the 1-particle RhoCI density.

Excited State 2: 2.147-A 2.6082 eV 475.37 nm f=0.0090 <S**2>=0.902
136A ->137A 0.47033
132B ->136B -0.21165
134B ->136B 0.81478

Excited State 3: 2.182-A 2.6389 eV 469.83 nm f=0.0314 <S**2>=0.940
135A ->137A 0.11589
136A ->137A 0.74494
129B ->136B -0.23895
132B ->136B -0.15991
134B ->136B -0.53013

Excited State 4: 2.338-A 2.9417 eV 421.47 nm f=0.0046 <S**2>=1.117
135A ->138A -0.11919
136A ->138A 0.95373
135B ->138B 0.18902

Excited State 5: 2.113-A 3.0313 eV 409.01 nm f=0.0022 <S**2>=0.866
135A ->139A 0.10204
136A ->139A 0.87001
130B ->136B -0.31888
131B ->136B -0.24600
132B ->136B -0.14750

Excited State 6: 2.128-A 3.1112 eV 398.52 nm f=0.0143 <S**2>=0.882
136A ->137A -0.15311
136A ->139A -0.14464
136A ->140A -0.24146
136A ->141A -0.13049
128B ->136B 0.18315
132B ->136B -0.19375
133B ->136B 0.85595

Excited State 7: 2.170-A 3.1344 eV 395.56 nm f=0.0007 <S**2>=0.928
135A ->138A 0.10026
136A ->140A 0.66181
130B ->136B -0.32902
131B ->136B 0.54754
133B ->136B 0.27021

Excited State 8: 2.431-A 3.2033 eV 387.06 nm f=0.0563 <S**2>=1.227
134A ->138A 0.18153
135A ->144A -0.10171
136A ->137A 0.26566
136A ->140A -0.16265
136A ->141A -0.20466
136A ->144A 0.13389
128B ->136B 0.28913
129B ->136B 0.10195
132B ->136B 0.69289
133B ->136B 0.10252
134B ->136B 0.13584
134B ->138B 0.19731
135B ->144B -0.11793

Excited State 9: 2.391-A 3.2303 eV 383.81 nm f=0.0222 <S**2>=1.179
129A ->137A 0.14861
132A ->137A 0.12693
135A ->141A -0.11456
136A ->140A -0.18955
136A ->141A -0.37214
136A ->143A -0.15804
128B ->136B 0.51674
129B ->137B -0.12927
130B ->136B -0.14550
131B ->136B 0.19102

131B ->140B 0.10644
132B ->136B -0.29540
132B ->137B -0.11016
133B ->136B -0.38784

Excited State 10: 2.682-A 3.2833 eV 377.62 nm f=0.0383 <S**2>=1.549
134A ->138A -0.31680
134A ->140A 0.10462
135A ->144A 0.13524
136A ->137A 0.17985
136A ->139A 0.30895
136A ->140A -0.10680
136A ->144A -0.18345
129B ->136B 0.32961
130B ->136B 0.39367
131B ->136B 0.34996
132B ->136B 0.19683
134B ->136B -0.13191
134B ->138B -0.34890
135B ->144B 0.14762

Excited State 11: 2.420-A 3.3119 eV 374.36 nm f=0.0413 <S**2>=1.214
134A ->138A 0.23718
136A ->137A -0.18431
136A ->139A 0.28191
136A ->140A -0.16545
136A ->144A 0.10856
129B ->136B -0.42491
130B ->136B 0.48614
131B ->136B 0.46290
134B ->138B 0.23105

Excited State 12: 2.157-A 3.3458 eV 370.56 nm f=0.0043 <S**2>=0.913
136A ->140A 0.58369
136A ->141A -0.20536
128B ->136B 0.20541
130B ->136B 0.55414
131B ->136B -0.43493

Excited State 13: 2.531-A 3.4555 eV 358.80 nm f=0.0312 <S**2>=1.351
134A ->138A 0.36568
134A ->140A -0.10927

136A ->137A 0.11795
129B ->136B 0.68565
130B ->136B 0.12420
131B ->136B 0.14157
132B ->136B -0.42931
134B ->138B 0.27252

Excited State 14: 2.582-A 3.6942 eV 335.62 nm f=0.0820 <S**2>=1.416
132A ->144A -0.10812
135A ->138A -0.20693
136A ->138A -0.10975
136A ->140A 0.11562
136A ->141A 0.71761
136A ->145A -0.14545
128B ->136B 0.33244
135B ->138B 0.29477

Excited State 15: 3.247-A 3.7721 eV 328.69 nm f=0.0110 <S**2>=2.386
128A ->137A 0.11722
130A ->139A 0.23238
130A ->140A 0.15420
131A ->139A -0.23666
131A ->140A 0.26186
135A ->137A -0.34584
136A ->137A 0.13882
136A ->142A -0.13380
128B ->137B -0.16092
129B ->136B -0.31701
130B ->139B -0.21268
130B ->140B -0.15893
131B ->139B 0.22916
131B ->140B -0.24891
132B ->136B -0.19487
135B ->137B 0.34816

Excited State 16: 3.041-A 3.8125 eV 325.21 nm f=0.0018 <S**2>=2.062
129A ->137A -0.12205
130A ->139A -0.23573
130A ->140A -0.15305
131A ->139A -0.11042
131A ->140A 0.15150
132A ->137A -0.11509

135A ->138A -0.28377
136A ->138A -0.14968
136A ->140A 0.10549
136A ->141A -0.32882
136A ->142A -0.24145
136A ->143A 0.15057
128B ->136B 0.22615
129B ->137B 0.12108
130B ->139B 0.22908
130B ->140B 0.14439
131B ->139B 0.10095
131B ->140B -0.15145
132B ->137B 0.10432
135B ->138B 0.48885

Excited State 17: 2.302-A 3.8333 eV 323.44 nm f=0.0037 <S**2>=1.075
131A ->139A -0.10381
131A ->140A 0.13297
136A ->142A 0.90175
128B ->136B 0.17071
131B ->140B -0.12993

Excited State 18: 2.852-A 3.8460 eV 322.37 nm f=0.0070 <S**2>=1.783
130A ->139A -0.20967
130A ->140A -0.12940
131A ->139A -0.11922
131A ->140A 0.16170
135A ->138A 0.24109
135A ->140A -0.10140
136A ->138A 0.15020
136A ->141A 0.26927
136A ->142A -0.26508
136A ->143A -0.11389
128B ->136B 0.48035
129B ->137B 0.11192
130B ->139B 0.18425
130B ->140B 0.10890
131B ->140B -0.13921
135B ->138B -0.44291

Excited State 19: 3.274-A 3.9584 eV 313.22 nm f=0.0025 <S**2>=2.429
133A ->138A 0.54171

133A ->140A -0.15711
133A ->145A 0.10125
133A ->146A 0.16155
134A ->138A -0.11639
136A ->144A 0.41227
133B ->138B -0.51851
133B ->140B 0.12437
133B ->145B -0.15693
134B ->138B -0.23590
135B ->144B -0.13006

Excited State 20: 2.959-A 3.9837 eV 311.23 nm f=0.0063 <S**2>=1.939
132A ->138A -0.10566
133A ->138A -0.35378
133A ->140A 0.10441
133A ->146A -0.10830
134A ->138A -0.12755
135A ->144A -0.13353
136A ->144A 0.65723
132B ->138B 0.14708
133B ->138B 0.32608
133B ->145B 0.11457
134B ->138B -0.32734
135B ->144B -0.18763

Excited State 21: 2.266-A 3.9924 eV 310.55 nm f=0.0171 <S**2>=1.033
136A ->143A 0.90553
136A ->145A -0.10908
128B ->136B 0.15411
135B ->138B -0.16612

Excited State 22: 3.347-A 4.1354 eV 299.81 nm f=0.0022 <S**2>=2.550
130A ->139A -0.22888
130A ->140A -0.14623
131A ->139A 0.18801
131A ->140A -0.19717
135A ->137A -0.31674
130B ->139B 0.21098
130B ->140B 0.14865
131B ->139B -0.18205
131B ->140B 0.18423
135B ->137B 0.73280

Excited State 23: 3.054-A 4.2525 eV 291.56 nm f=0.0188 <S**2>=2.082
127A ->138A 0.15039
129A ->144A -0.13634
132A ->144A 0.16697
134A ->144A 0.13993
134A ->146A -0.16518
135A ->141A 0.18732
135A ->145A -0.17819
136A ->141A 0.14475
136A ->143A 0.17264
136A ->145A 0.34566
127B ->138B -0.17466
128B ->136B 0.23559
129B ->144B -0.14545
132B ->138B 0.10126
132B ->144B 0.20091
134B ->145B -0.10785
135B ->138B 0.30901
135B ->141B -0.30192
135B ->143B -0.22441
135B ->145B 0.16438
135B ->146B -0.10292

Excited State 24: 2.232-A 4.2667 eV 290.59 nm f=0.0845 <S**2>=0.995
135A ->137A 0.77041
126B ->136B -0.11097
135B ->137B 0.54436

Excited State 25: 3.333-A 4.3313 eV 286.25 nm f=0.0029 <S**2>=2.528
129A ->138A -0.10553
129A ->140A 0.13342
130A ->137A 0.12890
131A ->137A -0.10723
132A ->138A 0.25958
134A ->138A 0.10630
135A ->139A -0.36973
136A ->144A 0.18868
136A ->146A -0.13023
128B ->139B -0.14349
129B ->140B -0.13420
132B ->138B -0.26516

135B ->138B 0.12610
135B ->139B 0.61079

Excited State 26: 2.178-A 4.3441 eV 285.41 nm f=0.0036 <S**2>=0.936
135A ->138A -0.43465
127B ->136B 0.80038
134B ->144B -0.16886
135B ->138B -0.23590

Excited State 27: 3.192-A 4.3766 eV 283.29 nm f=0.0013 <S**2>=2.297
129A ->138A 0.26810
130A ->137A 0.10103
130A ->138A 0.10015
131A ->138A 0.12545
132A ->138A -0.29307
132A ->140A 0.16745
134A ->138A -0.22356
135A ->138A -0.10580
135A ->139A -0.21035
135A ->144A -0.11761
135A ->146A -0.10746
136A ->144A -0.35828
136A ->146A 0.14808
129B ->138B -0.24378
131B ->138B -0.10792
132B ->138B 0.29121
132B ->140B -0.16050
135B ->139B 0.40338
135B ->144B -0.17057

Excited State 28: 3.236-A 4.4032 eV 281.58 nm f=0.0004 <S**2>=2.368
129A ->137A 0.10407
129A ->139A 0.11654
130A ->137A 0.13864
131A ->137A 0.15585
132A ->137A 0.27654
134A ->137A 0.62402
135A ->140A -0.22660
127B ->136B 0.10146
129B ->137B -0.13023
129B ->139B -0.13646
130B ->137B -0.11954

131B ->137B -0.12788
132B ->137B -0.21299
132B ->139B -0.10367
135B ->140B 0.31744

Excited State 29: 3.200-A 4.4179 eV 280.64 nm f=0.0005 <S**2>=2.310
129A ->139A -0.11390
130A ->137A -0.13624
131A ->137A -0.20423
132A ->139A -0.16822
134A ->137A 0.62904
135A ->138A 0.17152
135A ->140A 0.30145
128B ->140B 0.13206
129B ->139B 0.12900
130B ->137B 0.10787
131B ->137B 0.15058
132B ->139B 0.17059
135B ->140B -0.42764

Excited State 30: 3.006-A 4.4544 eV 278.34 nm f=0.0006 <S**2>=2.009
129A ->137A -0.29765
130A ->139A 0.14801
131A ->139A 0.10755
131A ->140A -0.13271
132A ->137A -0.29571
134A ->137A 0.33507
135A ->138A 0.24845
135A ->140A -0.12914
136A ->143A -0.14491
136A ->145A -0.10040
125B ->136B 0.26986
127B ->136B 0.12363
129B ->137B 0.24153
130B ->139B -0.13149
131B ->140B 0.11478
132B ->137B 0.25262
134B ->137B -0.17003
134B ->144B 0.10547
135B ->138B 0.13841
135B ->140B 0.23009

Ph$_2$PyIDBTM (UB3LYP/6-31G(d))

Excited State 1: 2.076-A 2.2795 eV 543.91 nm f=0.0983 <S**2>=0.828

159B ->160B 0.98220

This state for optimization and/or second-order correction.

Total Energy, E(TD-HF/TD-DFT) = -3334.70280711

Copying the excited state density for this state as the 1-particle RhoCI density.

Excited State 2: 2.236-A 2.4288 eV 510.47 nm f=0.0663 <S**2>=1.000

157A ->163A -0.10618

159A ->161A 0.12720

160A ->161A 0.86880

149B ->160B -0.11804

157B ->160B 0.35485

Excited State 3: 2.129-A 2.6764 eV 463.25 nm f=0.0078 <S**2>=0.884

158A ->162A 0.10967

152B ->160B -0.10939

158B ->160B 0.96358

158B ->162B -0.10720

Excited State 4: 2.346-A 2.8754 eV 431.18 nm f=0.0220 <S**2>=1.125

157A ->161A -0.10868

159A ->162A -0.12566

160A ->162A 0.85929

160A ->163A 0.21070

155B ->160B -0.15876

156B ->160B 0.20911

157B ->160B -0.16176

159B ->162B 0.16801

Excited State 5: 2.384-A 2.8958 eV 428.16 nm f=0.0518 <S**2>=1.171

157A ->161A -0.18976

159A ->163A 0.11677

160A ->161A 0.17258

160A ->162A -0.41696

160A ->163A 0.44146

148B ->160B -0.14330

154B ->160B 0.13036

155B ->160B -0.25756

156B ->160B 0.39008

157B ->160B -0.40736

157B ->161B 0.18068

Excited State 6: 2.239-A 2.9453 eV 420.96 nm f=0.2873 <S**2>=1.004

157A ->161A -0.10445

159A ->161A 0.10741

160A ->161A -0.37870

160A ->163A 0.25139

149B ->160B -0.12922

155B ->160B -0.25718

156B ->160B 0.26971

157B ->160B 0.71356

Excited State 7: 2.141-A 3.0412 eV 407.68 nm f=0.0278 <S**2>=0.896

160A ->164A -0.48328

160A ->165A 0.15987

153B ->160B 0.31009

154B ->160B 0.71212

155B ->160B 0.22971

157B ->160B 0.14417

Excited State 8: 2.143-A 3.0711 eV 403.71 nm f=0.0007 <S**2>=0.898

160A ->163A -0.11198

160A ->164A -0.30107

160A ->165A -0.32271

153B ->160B 0.73860

154B ->160B -0.39005

155B ->160B -0.20343

Excited State 9: 2.098-A 3.2189 eV 385.18 nm f=0.0097 <S**2>=0.851

160A ->163A -0.11716

154B ->160B -0.20734

155B ->160B 0.64071

156B ->160B 0.69617

Excited State 10: 2.199-A 3.2619 eV 380.10 nm f=0.0112 <S**2>=0.959

158A ->162A 0.13768

160A ->164A 0.74187

152B ->160B -0.12125

153B ->160B 0.47935

154B ->160B 0.31866

158B ->162B -0.12431

Excited State 11: 3.052-A 3.2725 eV 378.86 nm f=0.0048 <S**2>=2.078

151A ->162A -0.13620

158A ->162A -0.44942

159A ->168A 0.18855

160A ->164A 0.25340

160A ->168A -0.25409

152B ->160B 0.39814

152B ->162B 0.13228

153B ->160B 0.10105

158B ->160B 0.21204

158B ->162B 0.45840

159B ->168B 0.20887

Excited State 12: 2.141-A 3.3584 eV 369.18 nm f=0.0234 <S**2>=0.896

160A ->163A 0.22932

160A ->165A 0.85034

153B ->160B 0.25886

154B ->160B -0.31248

Excited State 13: 2.163-A 3.4252 eV 361.98 nm f=0.1156 <S**2>=0.920

160A ->163A 0.69121

160A ->165A -0.24850

160A ->170A 0.12774

152B ->160B -0.10049

154B ->160B -0.14271

155B ->160B 0.44616

156B ->160B -0.33762

159B ->162B -0.11450

Excited State 14: 2.655-A 3.4703 eV 357.27 nm f=0.0046 <S**2>=1.513

153A ->161A 0.11173

157A ->163A 0.13418

158A ->162A 0.33398

159A ->161A -0.18153

152B ->160B 0.77180

155B ->161B 0.10028

156B ->161B -0.10846

157B ->160B 0.10130

157B ->164B -0.11617

158B ->162B -0.24093

159B ->161B 0.11392

Excited State 15: 3.120-A 3.5253 eV 351.70 nm f=0.0253 <S**2>=2.183

150A ->166A 0.10921

152A ->167A 0.17350

153A ->161A -0.18429

155A ->165A 0.12722

157A ->163A -0.24774

158A ->162A 0.15568

159A ->161A 0.37104

149B ->160B 0.18089

150B ->166B -0.10833

151B ->167B 0.17336

152B ->160B 0.37044

154B ->165B -0.11349

155B ->161B -0.16643

156B ->161B 0.16332

157B ->160B -0.25135

157B ->161B -0.11661

157B ->164B 0.21625

158B ->162B -0.11554

159B ->161B -0.33124

Excited State 16: 3.144-A 3.5628 eV 347.99 nm f=0.0024 <S**2>=2.220

150A ->166A -0.18167

152A ->167A 0.11523

153A ->163A 0.19453

154A ->164A -0.16947

154A ->165A -0.10239

155A ->165A 0.11348

157A ->161A 0.39635

159A ->163A -0.16618

160A ->163A 0.18549

148B ->160B -0.11926

150B ->166B 0.17753

151B ->167B 0.11181

153B ->163B -0.15864

154B ->160B 0.12199

154B ->164B -0.10159

155B ->160B -0.27334

155B ->164B 0.12197

156B ->160B 0.25619

156B ->161B -0.10923

156B ->164B -0.15165

157B ->161B -0.38822

159B ->164B 0.12127

Excited State 17: 2.832-A 3.6761 eV 337.27 nm f=0.0016 <S**2>=1.755

150A ->166A -0.11540

152A ->167A -0.10004

153A ->161A 0.10843

154A ->164A 0.19170

154A ->165A 0.10133

155A ->165A 0.15956

159A ->161A 0.19652

160A ->161A -0.10452

160A ->169A -0.14469

148B ->161B -0.13482

149B ->160B -0.43347

150B ->166B 0.11839

151B ->160B 0.52484

151B ->167B -0.10484

153B ->163B 0.18680

154B ->165B -0.14810

157B ->160B -0.15773

159B ->161B -0.17202

Excited State 18: 2.485-A 3.6798 eV 336.93 nm f=0.0002 <S**2>=1.293

154A ->164A -0.11342

155A ->165A -0.10888

159A ->161A -0.11837

149B ->160B 0.28729

151B ->160B 0.80947

151B ->161B -0.13139

153B ->163B -0.11067

154B ->165B 0.10014

159B ->161B 0.10790

Excited State 19: 2.739-A 3.7349 eV 331.96 nm f=0.0006 <S**2>=1.626

149A ->161A 0.12063

150A ->166A -0.10411

154A ->164A 0.13489

155A ->165A -0.12246

159A ->162A -0.16880

160A ->166A -0.11315

148B ->160B -0.27105

149B ->161B -0.12505

150B ->160B 0.68033

150B ->161B 0.11512

150B ->166B 0.10591

153B ->163B 0.12747

154B ->165B 0.11652

159B ->162B 0.26198

Excited State 20: 2.771-A 3.7364 eV 331.82 nm f=0.0007 <S**2>=1.670

149A ->161A -0.10702

154A ->164A -0.11678

155A ->165A 0.11079

159A ->162A 0.22404

160A ->162A 0.12486

160A ->166A -0.10288

148B ->160B 0.24148

149B ->161B 0.11236

150B ->160B 0.66297

150B ->161B 0.11314

153B ->163B -0.11081

154B ->165B -0.10533

159B ->162B -0.34246

Excited State 21: 3.180-A 3.7395 eV 331.55 nm f=0.0022 <S**2>=2.278

149A ->161A -0.13078

152A ->167A -0.11635

153A ->162A -0.10539

154A ->164A -0.13443

155A ->164A -0.11040

155A ->165A 0.14542

159A ->162A -0.37111

160A ->162A -0.18580

160A ->163A 0.17917

160A ->165A -0.13674

160A ->170A -0.10974

148B ->160B 0.25134

149B ->161B 0.12210

151B ->167B -0.11592

153B ->163B -0.13763

154B ->163B -0.10150

154B ->165B -0.14062

159B ->162B 0.54695

Excited State 22: 2.684-A 3.9237 eV 315.99 nm f=0.0098 <S**2>=1.551

156A ->162A -0.11243

158A ->162A -0.17196

159A ->168A -0.15911

160A ->168A 0.78086

152B ->160B 0.17200

152B ->162B -0.13697

158B ->162B 0.40027

159B ->168B -0.19712

Excited State 23: 2.576-A 3.9761 eV 311.82 nm f=0.1152 <S**2>=1.409

154A ->164A 0.11415

159A ->161A 0.25308

149B ->160B -0.17749

159B ->161B 0.87824

Excited State 24: 3.437-A 3.9823 eV 311.34 nm f=0.0005 <S**2>=2.704

156A ->162A 0.64985

156A ->173A 0.20326

160A ->168A 0.15468

154B ->162B 0.12730

155B ->162B -0.40623

155B ->173B -0.13820

156B ->162B -0.44368

156B ->173B -0.14525

Excited State 25: 2.173-A 4.0025 eV 309.77 nm f=0.0017 <S**2>=0.931

157A ->166A -0.10046

160A ->166A 0.88873

160A ->167A -0.17307

160A ->170A -0.22891

160A ->171A -0.15837

150B ->160B 0.17341

Excited State 26: 2.234-A 4.0290 eV 307.73 nm f=0.0002 <S**2>=0.998

159A ->161A -0.10774

160A ->166A 0.18711

160A ->167A 0.81368

160A ->169A 0.30450

160A ->170A -0.12042

160A ->171A 0.22930

149B ->160B -0.10827

151B ->160B 0.11268

159B ->162B -0.10202

Excited State 27: 2.837-A 4.0688 eV 304.72 nm f=0.0915 <S**2>=1.762

150A ->166A -0.13095

152A ->167A -0.14199

153A ->161A 0.13687

154A ->164A -0.16339

155A ->165A -0.10816

159A ->161A 0.72587

160A ->166A -0.10337

160A ->167A 0.12685

160A ->170A -0.15656

149B ->160B 0.30051

150B ->166B 0.12973

151B ->167B -0.13631

153B ->163B -0.18055

154B ->165B 0.11612

Excited State 28: 2.817-A 4.0774 eV 304.08 nm f=0.0064 <S**2>=1.734

150A ->166A -0.10124

152A ->167A -0.12135

154A ->164A 0.23440

154A ->165A 0.11458

155A ->164A -0.16481

155A ->165A 0.25432

159A ->161A -0.18497

149B ->160B 0.68331

151B ->167B -0.10654

153B ->163B 0.23621

153B ->165B -0.10783

154B ->163B -0.11582

154B ->164B -0.10810

154B ->165B -0.23901

157B ->160B 0.13198

159B ->161B 0.11077

Excited State 29: 2.728-A 4.0928 eV 302.93 nm f=0.0042 <S**2>=1.610

151A ->168A -0.15761

155A ->165A -0.11386

159A ->161A 0.14484

159A ->163A -0.11685

159A ->172A -0.10809

160A ->166A 0.18167

160A ->167A 0.29324

160A ->169A -0.20347

160A ->170A 0.43273

160A ->172A 0.15623

160A ->173A 0.10884

160A ->174A -0.12675

148B ->160B 0.34638

152B ->168B -0.17386

154B ->165B 0.11012

159B ->162B 0.26433

159B ->164B 0.17753

159B ->170B 0.12071

159B ->172B -0.12673

Excited State 30: 2.305-A 4.1699 eV 297.33 nm f=0.0067 <S**2>=1.078

160A ->166A 0.18268

160A ->167A -0.17832

160A ->169A 0.25986

160A ->170A 0.61761

160A ->171A 0.42964

160A ->172A -0.13888

148B ->160B -0.33124

Mes_2PyIDBTM (UB3LYP/6-31G(d))

Excited State 1: 2.063-A 2.3605 eV 525.24 nm f=0.0879 <S**2>=0.814

183B ->184B 0.98511

This state for optimization and/or second-order correction.

Total Energy, E(TD-HF/TD-DFT) = -3570.61025951
Copying the excited state density for this state as the 1-particle RhoCI density.

Excited State 2: 2.214-A 2.7126 eV 457.07 nm f=0.0332 <S**2>=0.976
183A ->186A 0.12910
184A ->186A 0.73569
173B ->184B -0.18851
174B ->184B -0.27829
182B ->184B 0.49092

Excited State 3: 2.174-A 2.7470 eV 451.35 nm f=0.0198 <S**2>=0.931
182A ->185A -0.11572
184A ->186A -0.45641
173B ->184B 0.18299
182B ->184B 0.82642
182B ->185B -0.10371

Excited State 4: 2.350-A 2.8869 eV 429.48 nm f=0.0060 <S**2>=1.131
183A ->185A -0.13453
184A ->185A 0.95639
183B ->185B 0.20145

Excited State 5: 2.120-A 3.0682 eV 404.09 nm f=0.0024 <S**2>=0.873
184A ->187A -0.50887
184A ->188A 0.13043
175B ->184B 0.41560
176B ->184B 0.60021
177B ->184B 0.30719
180B ->184B -0.17971
181B ->184B 0.10135

Excited State 6: 2.069-A 3.0960 eV 400.47 nm f=0.0010 <S**2>=0.820
181B ->184B 0.98764

Excited State 7: 2.131-A 3.1005 eV 399.89 nm f=0.0013 <S**2>=0.885
184A ->187A -0.26495
184A ->188A -0.34283
175B ->184B 0.65464
176B ->184B -0.51897
177B ->184B -0.12146
178B ->184B -0.15818
180B ->184B 0.16427

Excited State 8: 2.071-A 3.1604 eV 392.31 nm f=0.0004 <S**2>=0.822
179B ->184B 0.99106

Excited State 9: 2.063-A 3.2112 eV 386.10 nm f=0.0006 <S**2>=0.814
184A ->188A 0.10170
176B ->184B 0.16452
177B ->184B 0.10259
180B ->184B 0.96378

Excited State 10: 2.295-A 3.2271 eV 384.20 nm f=0.0555 <S**2>=1.066
182A ->185A 0.12834
184A ->186A 0.23597
184A ->187A 0.19112
184A ->189A 0.20489
172B ->184B -0.27364
173B ->184B 0.12750
174B ->184B 0.35686
176B ->184B -0.24530
177B ->184B 0.64010
182B ->184B 0.11587
182B ->185B 0.13764

Excited State 11: 2.741-A 3.2697 eV 379.19 nm f=0.0292 <S**2>=1.629
173A ->186A 0.10742
182A ->185A 0.31350
183A ->192A 0.13194
184A ->186A 0.14879
184A ->188A -0.15426
184A ->189A -0.28063
184A ->192A -0.18102
172B ->184B 0.37039
174B ->184B 0.44798
175B ->187B -0.11170
177B ->184B -0.17079
182B ->184B 0.17948
182B ->185B 0.31243
183B ->192B -0.15726

Excited State 12: 2.070-A 3.2759 eV 378.47 nm f=0.0054 <S**2>=0.821
184A ->187A -0.37562
176B ->184B -0.16251

178B ->184B 0.88983

Excited State 13: 2.086-A 3.3073 eV 374.88 nm f=0.0114 <S**2>=0.837
184A ->187A 0.65272
175B ->184B 0.53154
176B ->184B 0.28697
178B ->184B 0.40352

Excited State 14: 2.744-A 3.3156 eV 373.94 nm f=0.0480 <S**2>=1.632
182A ->185A -0.33802
183A ->192A -0.11201
184A ->186A 0.18587
184A ->187A 0.12778
184A ->188A -0.23078
184A ->189A -0.24434
184A ->192A 0.16641
172B ->184B 0.35454
173B ->184B 0.27156
174B ->184B 0.12561
175B ->187B -0.11063
177B ->184B 0.36744
182B ->184B -0.12886
182B ->185B -0.33290
183B ->192B 0.13271

Excited State 15: 2.318-A 3.3457 eV 370.58 nm f=0.0885 <S**2>=1.093
182A ->185A 0.17172
184A ->186A -0.25833
184A ->188A -0.19893
184A ->189A -0.15212
172B ->184B 0.19365
173B ->184B -0.35444
174B ->184B -0.48743
176B ->184B -0.12670
177B ->184B 0.52820
182B ->185B 0.17987

Excited State 16: 2.185-A 3.4011 eV 364.54 nm f=0.0030 <S**2>=0.943
184A ->188A 0.81549
184A ->189A -0.21100
172B ->184B 0.19975
175B ->184B 0.23169

176B ->184B -0.33250
183B ->185B -0.10626

Excited State 17: 2.367-A 3.5547 eV 348.79 nm f=0.0333 <S**2>=1.150
182A ->185A 0.30618
184A ->186A 0.11541
173B ->184B 0.74089
174B ->184B -0.48907
182B ->185B 0.20567

Excited State 18: 3.469-A 3.6305 eV 341.51 nm f=0.0002 <S**2>=2.758
178A ->193A -0.40710
179A ->190A 0.43918
180A ->190A -0.28050
180A ->194A 0.11093
181A ->191A -0.16862
178B ->193B 0.40709
179B ->189B -0.52392
180B ->194B -0.13406
181B ->190B 0.17034

Excited State 19: 3.469-A 3.6310 eV 341.46 nm f=0.0000 <S**2>=2.758
178A ->193A -0.13416
179A ->190A 0.14421
179A ->194A -0.21564
180A ->194A -0.33671
181A ->191A 0.51029
178B ->193B 0.13389
179B ->189B -0.17282
180B ->194B 0.40727
181B ->190B -0.51916

Excited State 20: 3.034-A 3.7216 eV 333.15 nm f=0.0610 <S**2>=2.052
172A ->185A -0.10167
173A ->192A -0.11502
174A ->192A 0.14229
183A ->185A -0.38582
184A ->185A -0.20527
184A ->188A 0.14152
184A ->189A 0.39922
184A ->197A -0.12445
171B ->185B -0.10842

172B ->184B 0.22784
173B ->192B 0.10035
174B ->192B -0.12306
183B ->185B 0.58816
183B ->191B -0.12331

Excited State 21: 3.267-A 3.8290 eV 323.81 nm f=0.0086 <S**2>=2.419
172A ->186A -0.11415
175A ->187A -0.29432
175A ->188A -0.19052
176A ->187A 0.22932
176A ->188A -0.30503
183A ->186A 0.27812
184A ->186A -0.15039
184A ->192A 0.10568
172B ->186B 0.15249
173B ->184B 0.30086
174B ->184B 0.20351
175B ->187B 0.27272
175B ->188B 0.20000
176B ->187B -0.23063
176B ->188B 0.26968
177B ->188B 0.10731
183B ->186B -0.25386

Excited State 22: 2.852-A 3.8525 eV 321.83 nm f=0.0257 <S**2>=1.784
173A ->186A 0.10666
174A ->186A 0.12102
175A ->187A 0.19489
175A ->188A 0.12081
176A ->187A 0.13085
176A ->188A -0.17895
183A ->185A 0.20899
184A ->185A 0.12091
184A ->189A 0.65580
175B ->187B -0.21052
175B ->188B -0.12302
176B ->187B -0.12438
176B ->188B 0.18964
183B ->185B -0.40895

Excited State 23: 2.840-A 3.8857 eV 319.08 nm f=0.0194 <S**2>=1.766

175A ->187A -0.28191
175A ->188A -0.17417
176A ->187A -0.16895
176A ->188A 0.23071
183A ->185A 0.11182
184A ->189A 0.24690
184A ->197A -0.12843
172B ->184B 0.61722
173B ->186B 0.11266
174B ->186B 0.11831
175B ->187B 0.25249
175B ->188B 0.14739
176B ->187B 0.12767
176B ->188B -0.20140
183B ->185B -0.22545

Excited State 24: 2.693-A 3.9315 eV 315.36 nm f=0.0103 <S**2>=1.563
177A ->185A 0.11820
182A ->185A 0.16063
183A ->192A -0.13662
184A ->191A -0.11710
184A ->192A 0.76091
173B ->184B -0.18022
174B ->184B 0.11613
177B ->185B -0.11707
182B ->185B 0.39787
183B ->192B 0.19753

Excited State 25: 3.435-A 3.9901 eV 310.73 nm f=0.0005 <S**2>=2.699
176A ->185A -0.11102
177A ->185A 0.64689
177A ->197A 0.13822
177A ->198A 0.17933
184A ->192A -0.15546
174B ->185B 0.10174
176B ->185B 0.18806
177B ->185B -0.58268
177B ->197B -0.19960

Excited State 26: 2.093-A 4.1665 eV 297.57 nm f=0.0048 <S**2>=0.845
184A ->193A 0.13117
184A ->194A 0.23821

184A ->195A 0.93719

Excited State 27: 2.111-A 4.2290 eV 293.17 nm f=0.0005 <S**2>=0.864
184A ->190A 0.98259

Excited State 28: 3.396-A 4.2488 eV 291.81 nm f=0.0004 <S**2>=2.633
175A ->187A -0.20567
175A ->188A -0.12763
176A ->187A 0.14450
176A ->188A -0.18500
183A ->186A -0.46779
172B ->186B -0.10860
175B ->187B 0.19280
175B ->188B 0.13473
176B ->187B -0.14661
176B ->188B 0.16074
183B ->186B 0.66455

Excited State 29: 2.872-A 4.2606 eV 291.00 nm f=0.0112 <S**2>=1.812
171A ->185A 0.13074
173A ->192A -0.13424
174A ->185A 0.11738
174A ->192A 0.16556
182A ->192A 0.13016
182A ->198A 0.14182
183A ->185A -0.12263
183A ->189A -0.18487
183A ->197A 0.16515
184A ->189A -0.20195
184A ->196A 0.42162
184A ->197A -0.33189
171B ->185B -0.15808
172B ->184B -0.17190
173B ->192B 0.14838
174B ->185B -0.11261
174B ->192B -0.19125
182B ->197B 0.11754
183B ->185B -0.28507
183B ->186B -0.10552
183B ->191B -0.30826
183B ->197B -0.11968
183B ->198B -0.12932

Excited State 30: 2.117-A 4.2971 eV 288.53 nm f=0.0004 <S**2>=0.870
184A ->191A 0.97571
184A ->192A 0.10831

References.

[1] Ai, X., Chen, Y., Feng, Y., Li, F. A Stable Room-Temperature Luminescent Biphenylmethyl Radical. *Angew. Chem.Int. Ed.* **2018**, 57(11), 2869-2873.

[2] Abdurahman, A., Chen, Y., Ai, X., Ablikim, O., Gao, Y., Dong, S., Li, F. A pure red luminescent β-carboline-substituted biphenylmethyl radical: Photophysics, stability and OLEDs. *J. Mater. Chem. C* **2018**, 6(42), 11248-11254.

Disclaimer/Publisher's Note: The statements, opinions and data contained in all publications are solely those of the individual author(s) and contributor(s) and not of MDPI and/or the editor(s). MDPI and/or the editor(s) disclaim responsibility for any injury to people or property resulting from any ideas, methods, instructions or products referred to in the content.
